# Gene Profiling Characteristics of Radioadaptive Response in AG01522 Normal Human Fibroblasts

**DOI:** 10.1371/journal.pone.0123316

**Published:** 2015-04-17

**Authors:** Jue Hou, Fan Wang, Peizhong Kong, Peter K. N. Yu, Hongzhi Wang, Wei Han

**Affiliations:** 1 Center of Medical Physics and Technology, Hefei Institutes of Physical Science, Chinese Academy of Sciences, Hefei, China; 2 Department of Physics and Materials Science, City University of Hong Kong, Tat Chee Avenue, Kowloon Tong, Hong Kong; 3 Department of Radiation Oncology, First Affiliated Hospital, Anhui Medical University, Hefei, China; 4 Cancer Hospital, Hefei Institutes of Physical Science, Chinese Academy of Sciences, Hefei, China; Department of Genetics and Complex Diseases, UNITED STATES

## Abstract

Radioadaptive response (RAR) in mammalian cells refers to the phenomenon where a low-dose ionizing irradiation alters the gene expression profiles, and protects the cells from the detrimental effects of a subsequent high dose exposure. Despite the completion of numerous experimental studies on RAR, the underlying mechanism has remained unclear. In this study, we aimed to have a comprehensive investigation on the RAR induced in the AG01522 human fibroblasts first exposed to 5 cGy (priming dose) and then followed by 2 Gy (challenge dose) of X-ray through comparisons to those cells that had only received a single 2 Gy dose. We studied how the priming dose affected the expression of gene transcripts, and to identify transcripts or pathways that were associated with the reduced chromosomal damages (in terms of the number of micronuclei) after application of the challenging dose. Through the mRNA and microRNA microarray analyses, the transcriptome alteration in AG01522 cells was examined, and the significantly altered genes were identified for different irradiation procedures using bioinformatics approaches. We observed that a low-dose X-ray exposure produced an alert, triggering and altering cellular responses to defend against subsequent high dose-induced damages, and accelerating the cell repair process. Moreover, the p53 signaling pathway was found to play critial roles in regulating DNA damage responses at the early stage after application of the challenging dose, particularly in the RAR group. Furthermore, microRNA analyses also revealed that cell communication and intercellular signaling transduction played important roles after low-dose irradiation. We conclude that RAR benefits from the alarm mechanisms triggered by a low-dose priming radation dose.

## Introduction

Radioadaptive response (RAR) was first observed in cultured human lymphocytes 30 years ago [1],. This protective phenomenon is initiated by a low radiation dose (called priming dose and usually less than 200 mSv), which will alter gene expression profiles in cells and tissues [2–4] to induce protection against a subsequent high radiation dose (called challenging dose) [1, 5]. RAR has been widely observed in mammalian systems [6–8] such as mouse embryo cells [9], Chinese hamster fibroblasts [10], rabbit lymphocytes [11], hepatoma cell lines [12], U1-Mel human malignant melanoma cells [13], mouse germ cells [14, 15] and rat bone marrow cells [16], and can reduce cytogenetic damages, enhance cell survival and reduce tumor incidence [17–21].

RAR did not only occur *in vitro* but also *in vivo*. For examples, RAR was observed in mouse models [14, 22] as well as in the zebrafish embryo model [23–29]. The mice pre-exposed to low-dose radiation showed reduced chromosomal damages upon exposure to high-dose radiation, with the extent of reduction depending on the dose rate and the interval between exposures. Similarly, zebrafish embryos pre-exposed to low-dose radiation or the low-dose irradiated embryo conditioned medium showed fewer apoptotic signals when they were exposed to a subsequent high-dose radiation.

Nevertheless, our understanding on the underlying mechanism(s) of RAR is still fragmented and incomplete. Sometimes, there were conflicting results from experiments using different cell types and irradiation procedures [30]. In general, it is believed that the repair processes are enhanced in the “primed” cells (those having exposed to a priming dose) when compared to the “unprimed” cells. Proteins such as p53, protein kinase C, p38-MAPK, PARP, NF-κB, AP-1, phospholipase Cg2 (PLCG2) and cytosolic epoxide hydrolase (EPHX2) had been proposed [3, 30–32] as potential radiation biomarkers or molecular targets for RAR. For example, the transcription factor HNF4A was suggested to respond to low-dose radiation [33], as it activated specific target genes in response to oxidative stress [34]. On the other hand, high-dose radiation promoted apoptosis to eliminate cells with considerable DNA and other damages, followed by promoted growth and survival [35]. Although radiation responses are results of multiple signaling pathways, the transcriptional responses usually involve p53 and NF-κB networks. For example, TP53 was involved in the response to high-dose radiation, which however showed less response after low-dose exposures [36–38].

Microarray analysis is one of the most comprehensive approaches to identify gene expression and has led to significant advances in the knowledge of radiation-induced responses. Until now, however, limited studies have been performed to assess the RAR at the transcriptional level. Previous researchers observed RAR in three human lymphoblastoid cell lines and associated TP53-related genes with RAR [39]. Other studies focused on the changes in gene expression following direct high- or low-dose radiation [35, 40, 41] or following the induction of bystander effects [42, 43]. However, there was insufficient systematic analysis in most of the studies. This prompted us to perform systematic studies in order to understand the underlying mechanisms for RAR.

The critical factors involved in RAR induction were the priming dose, challenging dose and time interval between the applications of the two doses. In general, RAR responses are rarely induced when the priming dose is over 200 mGy, and are almost never induced when it is over 500 mGy [44]. Prior to this research, we studied various combinations of doses and time intervals, and found that a combination of a priming dose of 5 cGy, a challenging dose of 2 Gy dose and a time interval of 12 could induce a significant RAR response in AG01522 cells. In the present study, we focused on mRNA and microRNA microarray studies, and aimed to characterize the transcriptome for RAR in AG01522 human skin fibroblasts and to examine the functional regulatory networks at the genetic level. AG01522 cells were exposed at a specific time point to a challenging dose of 2 Gy in the RAR group, or a priming dose of 5 cGy in the low-dose group. The micronucleus (MN) assay was also employed to quantify the radiation-induced chromosome aberrations. The results could provide useful information for identifying biomarkers of RAR, and thus help us better understand RAR itself and its potential implications on radiotherapy procedures.

## Materials and Methods

### Cell Culture

Human skin fibroblast AG01522 cells, was gifted by Dr. Kevin Prise (Centre for Cancer Research & Cell Biology, Queen's University Belfast, United Kingdom) which were obtained from the Coriell Cell Repositories at the Coriell Institute for Medical Research (Camden, NJ), previously employed by several research groups to demonstrate RAR [45], were used in our experiments. The AG01522 cells were cultured in modified α-MEM (Hyclone, Logan, US) supplemented with 20% fetal bovine serum (FBS) (Hyclone, Logan, US), 1% penicillin/streptomycin, 1% glutamine and 1% non-essential amino acids mix (Sigma-Aldrich, St. Louis, US). All cultures were grown in a humidified 5% CO_2_ atmosphere at 37°C. All procedures involving human cell line use were approved by the Hefei Institutes of Physical Science Committee (Chinese Academy of Sciences).

### X-ray Irradiation

Irradiation was provided by an X-ray radiation machine (XSZ-220/20, Kangjia and Dandong, China). Briefly, the cells were exposed to a priming dose of 5 cGy (60 kV, 4.2 mA and 10.5 cGy/min), or a challenging dose of 2 Gy (120 kV, 12.2 mA and 201.5 cGy/min), or a priming dose of 5 cGy followed by a challenge dose of 2 Gy (5 cGy + 2 Gy) 12 h later. The samples were then collected for subsequent experiments performed at 3, 6, 12 and 24 h after the last irradiation. Sham-irradiated samples covered with a lead mask were used as control samples.

### MN assay

MN tests were performed to assess the chromosome aberrations in each group. Briefly, the cells were trypsinized and digested at 3 h after irradiation, and 5×10^4^ cells were seeded in every well of the 6-well cell culture plate. Cytochalasin B (Sigma-Aldrich, St. Louis, US) was added into the culture medium with a final concentration of 2.5 μg/ml and the cells were then returned into the incubator. After 48 h, the cells were rinsed with PBS solution, fixed in 2% paraformaldehyde for 20 min, stained with acridine orange for 3 min and rinsed with PBS solution for 10 min. At least 1000 binucleate cells were examined and the number of cells with micronuclei was scored in each sample under a fluorescence microscope (Leica DMI 4000B, Germany). The frequency of cells with micronuclei (F_MN_) was calculated as: F_MN_ = the number of cells with micronuclei / the number of binucleate cells examined. Statistical analysis was performed on the means of the data obtained from three independent experiments. Statistical significance was assessed through t-rests or one-way ANOVA where appropriate.

### RNA Preparation

The collected cell pellets were lysed and extracted by TRizol (Life technologies, Carlsbad, CA, US) following the manufacturer’s instructions, and checked for RNA Integrity Number (RIN) to assess the RNA integrity by an Agilent Bioanalyzer 2100 (Agilent technologies, Santa Clara, CA, US).

Qualified total RNA was further purified using the RNeasy micro kit (QIAGEN, GmBH, Germany) and genomic contamination was removed by RNase-Free DNase Set (QIAGEN, GmBH, Germany). Small molecular RNA was extracted using the mirVana miRNA Isolation Kit (Ambion, Austin, TX, US) following the manufacturer’s instructions. The purified RNA was stored at—80°C.

### Microarray Hybridization

For Affymetrix PrimeView Human Gene Expression Chip:

The total RNA was amplified, labeled and purified using the GeneChip 3’IVT Express Kit (Affymetrix, Santa Clara, CA, US) following the manufacturer’s instructions to obtain biotin-labeled cRNA. After hybridization on Human PrimeView Arrays for 16 h at 45°C and 60 rpm in the Hybridization Oven 640 (Affymetrix, Santa Clara, CA, US), slides were washed and stained with a Fluidics Station 450 (Affymetrix, Santa Clara, CA, US). Scanning was performed on a seventh-generation GeneChip Scanner 3000 (Affymetrix, Santa Clara, CA, US). Affymetrix GCOS software was used for image analysis to generate raw intensity data.

For Agilent Human miRNA v19.0 Chip (Catalog ID: G4872A-046064):

Each miRNA v19.0 slide was hybridized with 100 ng Cy3-labeled RNA using the miRNA Complete Labeling and Hyb Kit (Agilent technologies, Santa Clara, CA, US) in the Hybridization Oven (Agilent technologies, Santa Clara, CA, US) at 55°C, 20 rpm for 20 h according to the manufacturer’s instructions. After hybridization, slides were washed with the Gene Expression Wash Buffer Kit (Agilent technologies, Santa Clara, CA, US). Slides were scanned by Agilent Microarray Scanner (Agilent technologies, Santa Clara, CA, US) using the Feature Extraction software 10.7 (Agilent technologies, Santa Clara, CA, US) with default settings.

### Microarray data analysis for differential expression gene (DEG)

All microarray data analysis was performed in R (http://www.r-project.org/) with multiplex packages involving Affymtriex and Angilent platforms for data analysis and statistical analysis in Bioconductor. The initial data quality was assessed by the background level, RNA quality, and pair-wise correlation among the samples.

For the PrimeView Chip, the customized CDF file (version 17, ENTREZG) downloaded from the BrainArray website was performed in probe set mapping [46, 47]. IQR was used for raw data filtering with the genefilter package; with the threshold set to remove intensities lower than 20% of the IQR global intensity. Normalization was performed with the RMA algorithm which included the global background adjustment and quantile normalization. Empirical Bayes moderation of the standard error and Benjamini and Hochberg false-discovery rate correction for multiple testing were employed, as implemented in the limma package [48]. Differentially expressed genes were identified with threshold fold changes of more than 1.2 and *p* values smaller than 0.05.

For the miRNA chip, the quality and comparability of samples before and after normalization were further checked by statistics, intensity boxplots and MA figures. The raw data were normalized with quantile normalization to ensure comparability across samples. The probes corresponding to the same miRNA were summarized for each sample by the median of the respective normalized expression values. To identify miRNAs that were differentially expressed, we applied the empirical Bayes moderated t-statistics implemented in the limma package (*p* < 0.05). Statistical models were fitted to the data, and comparisons of interest were extracted as contrasts. Unsupervised hierarchical clustering with complete linkage, using both Euclidean and (1-Pearson correlation) as distance metrics, was applied to cluster the samples according to their miRNA expression levels.

Venn diagrams were used to select the miRNAs specific to RAR. The three-way Venn diagram indicated the numbers of miRNA identified as significant (*p* < 0.05) from the 5 cGy, 2 Gy and (5 cGy + 2 Gy) groups. The numbers inside the intersections of circles denote the numbers of miRNAs significant for two or three of the groups.

### Gene set enrichment analysis (GSEA)

Gene set enrichment analyses (GSEA) for the samples from the 5 cGy, 2 Gy, (5 cGy + 2 Gy) groups and RAR samples were carried out using the GSEA software (http://www.broadinstitute.org/gsea/index.jsp) at the gene level (Collapse dataset to gene symbols = false) with 1,000 gene set permutations. The genes in the expression datasets were ranked using the Signal2Noise metric (comparison with each time point and baseline) and Pearson metric (time-course dataset) following GSEA recommendations for our sample sizes. Significance was defined at the False Discovery Rate (FDR) *q*-value = 0.25 level. Some results out of the FDR threshold, NOM *p*-value = 0.01, were considered alternatively. The Cytoscape (v 3.1.1) plugins Enrichment Map (v 2.0) [49] and Word Cloud (v 2.0.1) were used to visualize the GSEA networks. The cutoff for the overlap coefficient was set to 0.3, FDR *q*-value to 1 and *p*-value to 0.01.

### Gene ontology categories analysis

To link our data to prior knowledge we performed gene ontology categories analysis by using the Cytoscape (http://www.cytoscape.org/) plug-in BiNGO (v2.44)[50]. To include only significant results, the FDR threshold was set to 0.05.

### Pathway enrichment analysis

Pathway enrichment analysis was performed using the Cytoscape plug-in ClueGO (v2.1.1) and CluePedia (v1.1.1) [51, 52]. The KEGG, REACTOME and WikiPathways databases were employed. The Benjamini and Hochberg false-discovery rate was set to 0.05.

### qRT-PCR analysis of mRNA expression

To validate the differentially expressed mRNAs by real-time PCR, 1 μg of the total RNA was reverse-transcribed using the One Step PrimeScript RT-PCR Kit (TaKaRa, Japan). Quantitative RT-PCR was carried out by using a Light Cycler 480 II instrument (Roche, Indianapolis, IN). The primer sequences used for RT-PCR can be found in the S1 Table. Fold changes in gene expression were calculated using the ΔΔCt method.

### Western blot

In this experiment, AG01522 cells were exposed under each strategy and harvested at 12, 24 or 48 h post exposure. The cells were rinsed once with ice-cold PBS, centrifuged at 230 g for 5 min and lysed in ice-cold lysis buffer containing protease and phosphatase inhibitors (Thermo Fisher Scientific, Rockford, IL, USA) and then 2 mM phenylmethanesulfonyl fluoride (PMSF) was added. Subsequently, the cells were kept on ice for 30 min and vortexed every 10 min to ensure a complete cell lysis. Lysates were centrifuged at 10000 rpm. The supernatants were isolated and the protein concentration was adjusted in all samples prior to heating at 95°C for 5 min in 1× loading buffer (0.25 M Tris, 2% SDS, 10% glycerol, 2% β-mercaptoethanol, 0.004% bromphenolblue) and then subjected to SDS-PAGE (sodium dodecyl sulfate poly-acrylamide gel electrophoresis) on precast 12% PAGE gels. The proteins were blotted onto PVDF membranes. Subsequently, unspecific binding was blocked with 0.5% nonfat milk powder in Tris-buffered saline (20 mM Tris, 13.7 mM NaCl) containing 0.1% Tween (TBS-T) for 30 min. After this, the membrane was incubated with the corresponding primary antibody, p21/WAF1 (Millipore) (#05–345), Phospho-p38 MAPK (#4511) and Phospho-NF-κB p65 (#3036) (Cell Signaling, Beverly, MA, USA) with appropriate dilution at 4°C overnight. Incubation was followed by three washing steps with TBS-T and incubation with horseradish peroxidase-coupled secondary antibodies 1:1000 for 1 h at room temperature. After three washing steps with TBS-T, the membranes were visualized by using the Western Blotting Substrate system. The same membranes were stripped and relabeled with antibodies directed against the corresponding total protein as well as β-tubulin as a loading control.

### miRNA and their targets

Three web databases of miRNA target prediction were used in this research. One was the miRBase::Targets (release 5) database (http://microrna.sanger.ac.uk/targets), which used the miRanda algorithm to predict miRNA targets [53]. Another database was the TargetScan (release 4.1) (http://www.targetscan.org), which provided the prediction results computed by the TargetScanS algorithm [54, 55]. The third database was miRGen, which incorporated algorithms including TargetScan, miRanda and PicTar (http://www.diana.pcbi.upenn.edu/cgibin/miRGen).

### qRT-PCR analysis of miRNA expression

To validate the differentially expressed miRNAs by real-time PCR, 1 μg of small RNA was used for reverse transcription with the miScript II RT Kit (QIAGEN, GmBH, Germany). Quantitative RT-PCR was carried out using a Light Cycler 480 II instrument (Roche, Indianapolis, IN). The PCR primers for hsa-let-7b-3p, hsa-miR-1185-5p, hsa-miR-1236-5p, hsa-miR-222-5p, hsa-miR-3659 hsa-miR-4535, hsa-miR-492, hsa-miR-877-5p and U6 were synthesized and listed in S2 Table miScript SYBR Green PCR Kit (QIAGEN, GmBH, Germany) were used in the real time PCR reaction according to the manufacturer’s suggested protocols. The relative gene expression was calculated using the ΔΔCt method based on the U6 RNA levels.

## Results

### MN induction in RAR

**Fig 1:**
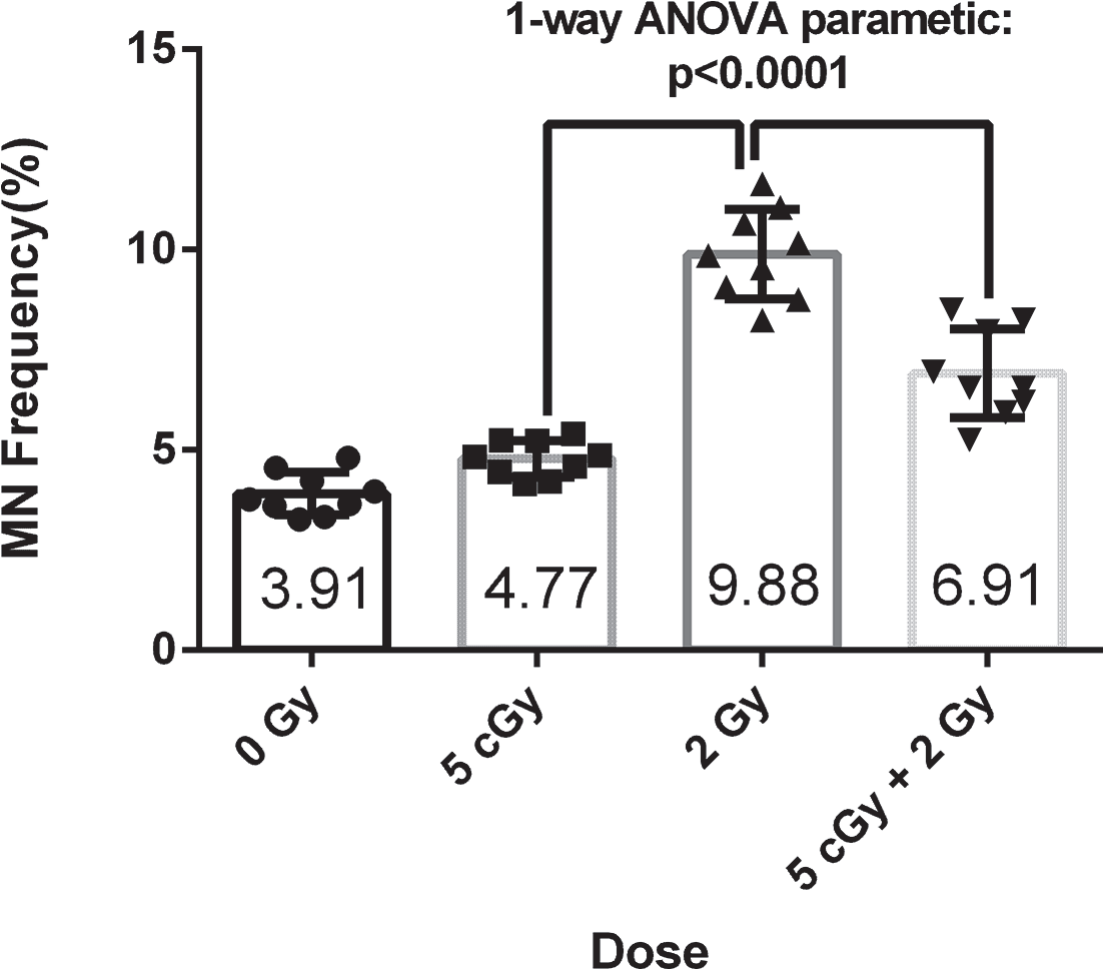
The RAR effect revealed by the micronucleus assay. The 0 Gy group was a sham control, without any radiation treatment. The 5 cGy and 2 Gy groups were irradiated with 5 cGy and 2 Gy doses, respectively. The (5 cGy + 2 Gy) group was irradiated with a priming dose of 5 cGy, followed by a challenging dose of 2 Gy, with a 12 h interval between application of the two doses.

As shown in Fig 1, a low-dose irradiation (5 cGy) slightly increased the MN yield (4.77%) compared with the control (3.91%) while a high-dose irradiation (2 Gy) significantly enhanced the MN yield (9.88%). However, a priming radiation dose of 5 cGy applied at 12 h beforehand reduced the amount of chromosomal aberrations induced by the subsequent challenging dose of 2 Gy by about one third. These results were similar to those reported previously [45], and confirmed significant RAR in this cell model. As such, this cell model could be applied in microarray profiling.

### Gene expression profiles

**Fig 2:**
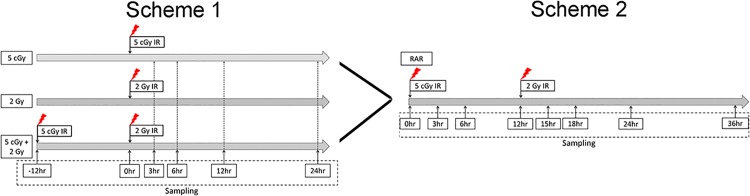
The microarray analysis timeline.

The gene profiles for the single dose groups i.e., those subjected to 5 cGy (top panel of Scheme 1 in Fig 2) or 2 Gy (middle panel of Scheme 1 in Fig 2) irradiation at different time points after irradiation, at 0 (baseline), 3, 6, 12 and 24 h after irradiation were examined). Similarly, the gene profiles for the (5 cGy + 2 Gy) groups only after application of the challenging dose were examined (illustrated in the bottom panel of Scheme 1 in Fig 2). Finally, the gene profiles for the RAR groups, i.e., those subjected to a priming dose of 5 cGy applied at four different time points (viz., 0, 3, 6 and 12 h) and then subjected to a challenging dose of 2 Gy at 12 h after the priming dose, were examined, the procedures of which are shown as Scheme 2 in Fig 2.

After exposures to the desired radiation doses, the numbers of differentially expressed genes reached a maximum at 24 h post irradiation (Table 1), with fewer genes responding at 6 or 12 h and no genes with FDR < 10% at 3 h. Due to the limited numbers or absence of genes significantly expressed at early time points for each group, we relaxed the FDR requirement for all data set analysis in the limma function, by choosing an unadjusted *p* value of 0.05 as the threshold. The heat maps of different expression genes are shown in S1 Fig.

**Table 1:** Summary of gene numbers under differential statistical thresholds.

		5 cGy				2 Gy				5 cGy + 2 Gy			
		3 h	6 h	12 h	24 h	3 h	6 h	12 h	24 h	3 h	6 h	12 h	24 h
Unadjp0.05	Up	99	152	369	815	669	170	346	976	234	279	542	1272
	Down	92	148	240	615	249	179	428	860	206	392	510	1195
	Total	191	300	609	1430	918	349	774	1836	440	671	1052	2467
Unadjp0.01	Up	10	37	100	315	139	55	125	403	61	100	167	628
	Down	16	33	51	235	45	31	147	390	57	105	184	608
	Total	26	70	151	550	184	86	272	793	118	205	351	1236
Unadjp0.005	Up	4	16	59	188	55	31	74	250	32	57	101	430
	Down	5	10	30	140	24	16	92	264	30	54	113	437
	Total	9	26	89	328	79	47	166	514	62	111	214	867
BH p0.1	Up	N/A	N/A	N/A	14	N/A	N/A	7	101	1	1	10	411
	Down	N/A	N/A	N/A	11	N/A	N/A	8	128	1	0	15	424
	Total	N/A	N/A	N/A	25	N/A	N/A	15	229	2	1	25	835
BH p0.05	Up	N/A	N/A	N/A	4	N/A	N/A	1	33	N/A	N/A	4	181
	Down	N/A	N/A	N/A	2	N/A	N/A	3	54	N/A	N/A	5	221
	Total	N/A	N/A	N/A	6	N/A	N/A	4	87	N/A	N/A	9	402

Furthermore, we also validated the gene profile results by qRT-PCR method for a number of genes. The qPCR results confirmed the microarray results in terms of the trends in gene changes (as shown in S3 Table). These data have been deposited in NCBI’s Gene Expression Omnibus and are accessible through GEO Series accession number GSE 59863 (mRNA profile data GSM number: GSM 1448246–1448285, miRNA profile data GSM number: GSM 1448296–1448307).

### Gene Set Enrichment Analysis

GSEA detects statistically significant differences in *a priori* defined gene sets (pathways) through a weighted Kolmogorov-Smirnov statistic (Normalized Enrichment Score (NES)) based on over-representation of gene-set members toward the top or bottom of a list of genes ranked by the strength of their correlation to the phenotypes (irradiation doses and groups).

Considering the time-course data, positive correlations indicated that the gene sets were enriched at later time points, while negative correlations indicated that the gene sets were enriched at earlier time points. For some groups, no gene sets were significant at FDR < 25%, in which case we chose the pathways or gene ontology (GO) terms with nominal *p* values < 1% for further analysis, as shown in Table 2 in detail.

**Table 2:** Summary of gene set numbers which were significantly enriched.

	Correlation	FDR < 25%	Nom p value < 1%	Nom p value < 5%
5 cGy	Positive	0	11	84
	Negative	0	38	188
2 Gy	Positive	0	3	43
	Negative	0	37	159
5 cGy + 2 Gy	Positive	0	11	62
	Negative	31	45	183
RAR	Positive	0	12	77
	Negative	91	48	176

**Fig 3:**
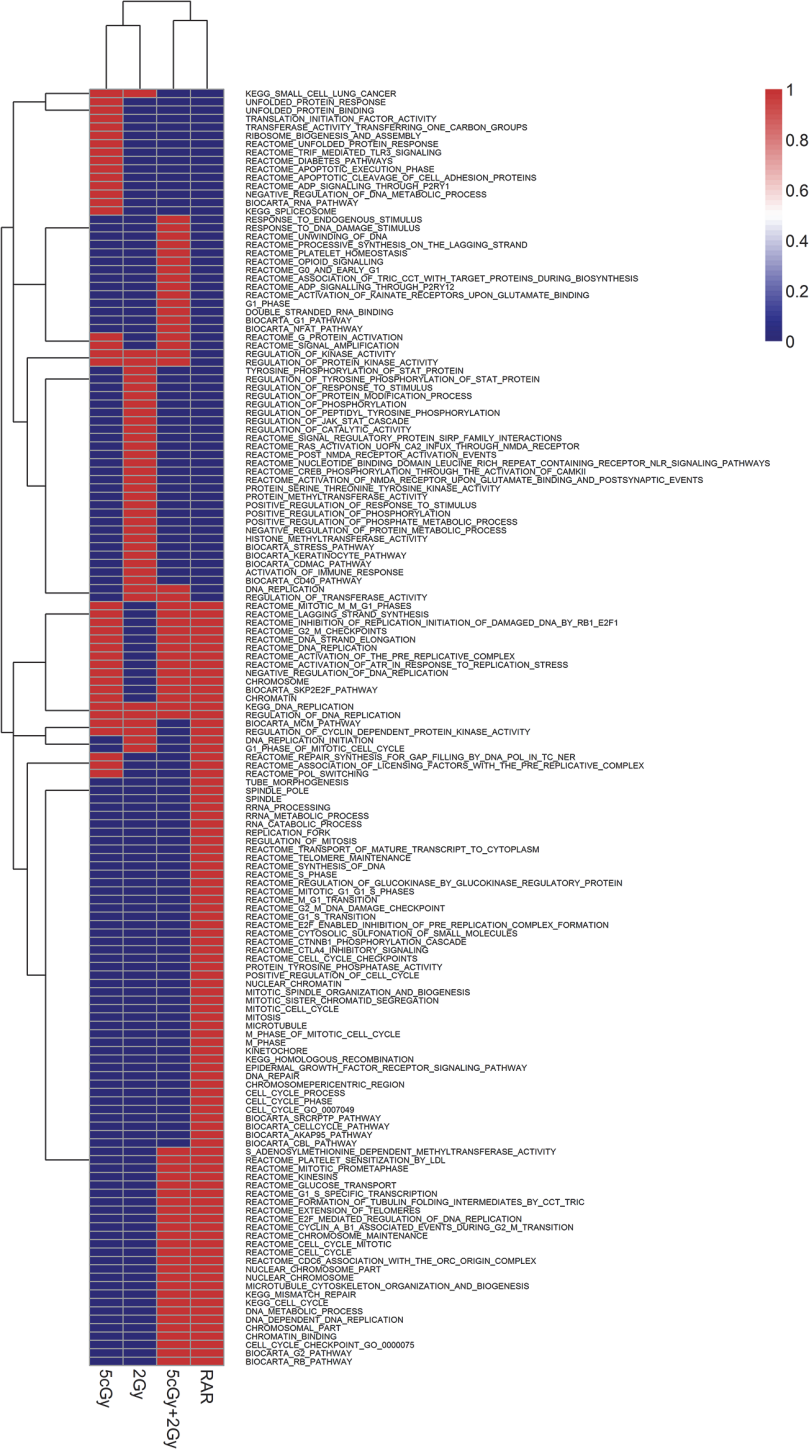
Clustering of GSEA results enriched with negative correlation with increased time profile. Sorting negative correlation The GSEA results were sorted by FDR < 25% or nom p value < 1% thresholds, where appropriate. The heatmap illustrated the gene sets for each irradiation group. The red color codes for the presence of the shown gene sets in a particular group, while the blue color codes for absence of the shown gene sets in a particular group.

**Fig 4:**
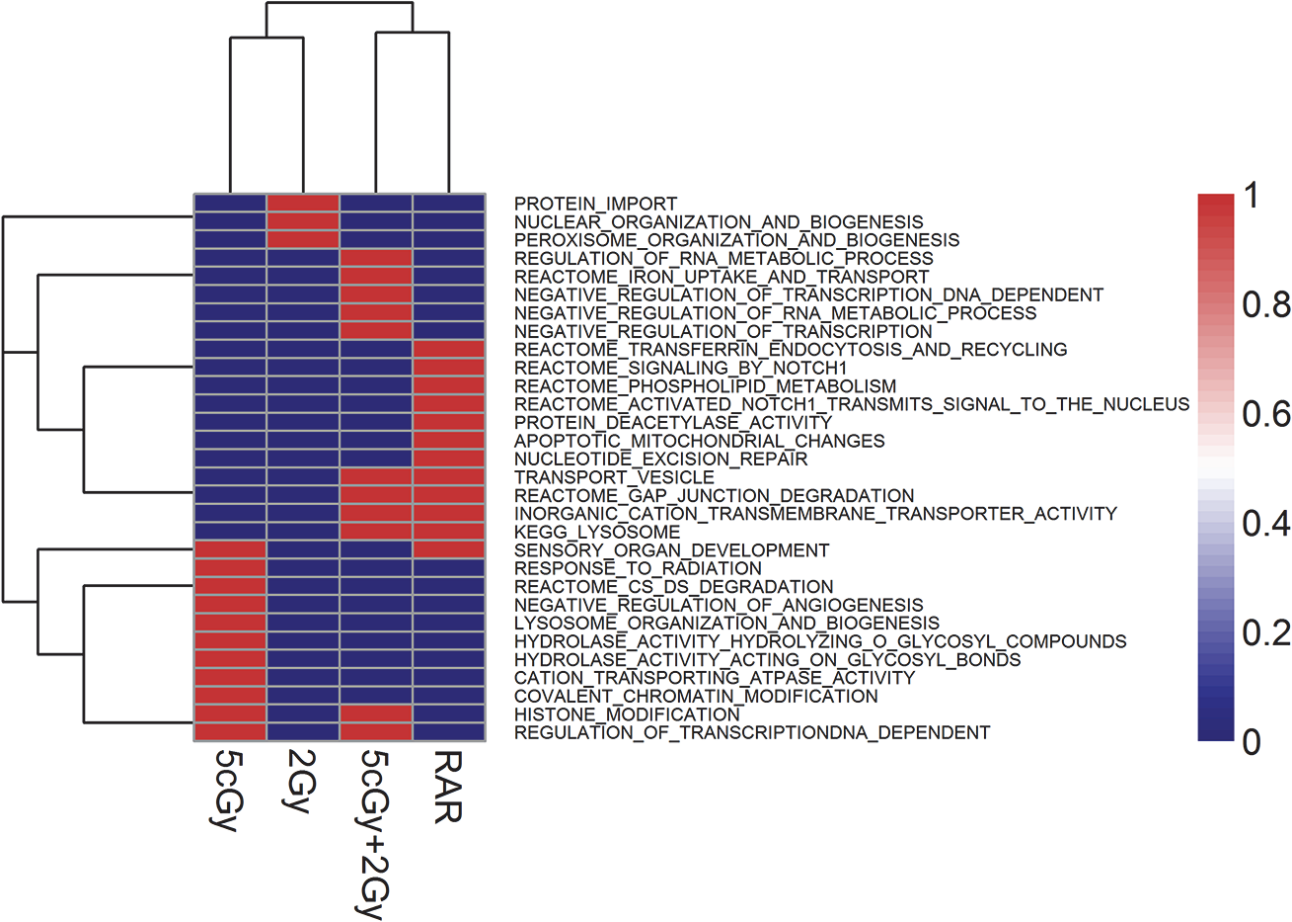
Clustering of GSEA results enriched with positive correlation with increased time profile, for various irradiation groups (viz. 5 cGy, 2 Gy, (5 cGy + 2 Gy) and RAR). The GSEA results were sorted by FDR < 25% or nom p value < 1% thresholds, where appropriate. The heatmap illustrated the gene sets for each irradiation group. The red color codes for the presence of the shown gene sets in a particular group, while the blue color codes for absence of the shown gene sets in a particular group.

In general, for the 5 cGy group, the enriched gene sets with negative correlations (Fig 3) involved unfolded protein response, apoptotic execution, spliceosome, DNA and RNA metabolic processes, G protein activation and signaling amplification, kinase activity, DNA repair and replication, and cell cycle arrest. These showed that the cells initiated many biological processes to respond to the stress within a short time after X-ray exposure. Moreover, the 5 cGy low-dose irradiation also induced gene sets or pathways for lysosome organization, hydrolase activity and histone modification, which led to repair of radiation, induced damages and enriched inclination to positive correlation with timeline (Fig 4). The enrichments with negative and positive correlations are presented in S2 Fig.

For the 2 Gy group, most gene sets enriched at early time points were negatively correlated with time-course phenotypes (Fig 3). These gene sets included kinase activity and regulation, phosphorylation and cascade of STAT, RAS, JAK-STAT, CREB transcription factors, regulation of cell cycle and DNA replication, and moreover, some responses to stress pathways or external stimuli. Only three terms involved nuclear and peroxisome organization biogenesis showed positive correlation with timeline (Fig 4), which suggested that the cells detoxicated various toxic substances induced by irradiation. In particular, peroxisomes were shown to generate superoxide (O_2_^•–^) and nitric oxide (^•^NO) radicals [56, 57]. The enrichments with negative and positive correlations are presented in S3 Fig.

For the (5 cGy + 2 Gy) group, response to DNA damage stimulus, G protein activation and signaling amplification, kinase activity, cell cycle, DNA replication and chromosome maintenance showed enrichments at early time points after the application of the 2 Gy challenging dose (Fig 3). However, the RNA metabolic process and transcriptional regulation were up-regulated sustainably. Similar to the 5 cGy group, the gene sets and pathways for lysosome and histone modification were present. Furthermore, some terms involving iron uptake and transport were also positively enriched (Fig 4). The enrichments with negative and positive correlations are presented in S4 Fig.

To further study the RAR effect, we combined part of the gene profiles for the 5 cGy group with those of the (5 cGy + 2 Gy) group, to provide more information or to show the variance in details. The Notch1 signaling pathway and nucleotide excision repair term were enriched in the positive correlation of RAR (shown in S5 Fig), while most of the gene sets were essentially comparable regardless of the positive or negative correlations.

These GSEA results revealed that the 5 cGy radiation could induce early cell response, alter the metabolism and biological process to defend against radiation-induced damages, and promote repair of the damages. However, the 2 Gy high-dose radiation tended to provoke survival and did not resist the damages or repair them rapidly, which might be detrimental to the basic biological functions of the cells.

### Investigation of radiation-induced gene expression alteration through GO analysis

Within any given gene sets, GO-based functional analysis provides statistically enriched GO terms, describing gene products and demonstrating their relationships according to three ontology categories: biological process, molecular function and cellular component. To analyze our microarray data based on groups of functionally related genes instead of individual genes, BiNGO was used as a Java-based high-throughput functional genomic analysis tool in Cytoscape. We then identified the significantly enriched GO terms and characterized the radiation responses to different radiation doses and their dynamic changes over time after exposures.

Low-dose radiation (5 cGy) could up-regulate G-protein coupled receptor activity and olfactory receptor activity through sensory perception of chemical stimulus. However, high-dose radiation (2 Gy) induced complex and extensive transmembrane receptor activities, mostly involving G-protein coupled receptor, olfactory receptor, and signal transducer activities in the downstream. In addition, more biological processes, especially the nucleotide metabolic processes were abundant. Interestingly, the (5 cGy + 2 Gy) group did not promote any up-regulation at 3 h, but a large number of genes involved in DNA metabolic processes, DNA replication, cellular responses to external stress or stimuli, and cell cycle showed down-regulation, suggesting the (5 cGy + 2 Gy) radiation scheme could negatively regulate cell processes, inhibit DNA replication and cell cycle arrest in the very early phase (data shown in S6 Fig).

At 6 h after exposure (data shown in S7 Fig), the 5 cGy group showed up-regulations of metal-ion-binding related genes or GO terms, which would be a sign that metal ions were required during DNA repair and replication. When we examined the 2 Gy radiation-induced gene expression profiles, some typical processes showed up, including apoptosis, PCNA-p21 activation, cell cycle arrest, DNA damage responses signal transduction by TP53. The GO terms revealed that the irradiated cells were repairing the radiation-induced damages, surviving or dying. Meanwhile, for the (5 cGy + 2 Gy) scheme, the cells assembled all resources to participate in the repair. Most biological processes concentrated on the negative regulation of DNA replication, transcription, and nucleotide metabolism, to prevent the fatal cascade reactions of DNA damages.

At 12 h post-radiation (data shown in S8 Fig), 5 cGy radiation induced responses dominated by positive regulation of ion binding, gene expression and cellular metabolism. Moreover, complicated cellular processes showed down-regulation, including responses to stimuli, cell proliferation, primary metabolism, and kinase phosphatase activity. At this time point, the cells having been exposed by 2 Gy radiation shifted their focus to negative regulation of DNA replication, which was largely similar to the characteristics of the cells exposed to (5 cGy + 2 Gy) radiation at the 6 h time point. Furthermore, for the (5 cGy + 2 Gy) strategy, in addition to down-regulation of DNA replication, cellular processes were initiated to control the cell cycle to give more time for repairing the cellular damages.

Up to 24 h after radiation exposure (data shown in S9 Fig), a wide range of GO terms were present. The cells irradiated with a 2 Gy dose finally caught up the progress of the cells which had been subjected to (5 cGy + 2 Gy) radiations. Both these groups showed cell cycle arrest, as well as negative regulation of cell cycle checkpoint and DNA replication. Nevertheless, activation or inhibition of similar biological processes in the 2 Gy group were initiated 6–12 h later than those in the (5 cGy + 2 Gy) group. This could be a critical reason explaining that the priming dose helped protect against damages induced by the challenging dose.

Furthermore, we assessed the protein expressions of p21/WAF1, phospho-p38 MAPK and phosphor-NF-κB through the Western Blot assay. The results (shown in S10 Fig) reflected that the p21 protein was up-regulated after irradiation at various time points, which was consistent with previous studies [58, 59]. Moreover, the levels of phosphorylated p38 MAPK and NF-κB were also increased at 24 h, which suggested the activation of the mediated signaling pathways.

Overall, the gene ontology results for each irradiation scheme appeared to be relatively similar at different time points. However, there were still observable differences. The GO terms of the 2 Gy group at all-time points showed that they were lagging behind those for the (5 cGy + 2 Gy) group, which suggested that the 5 cGy irradiation changed the cellular response to external stresses.

### Investigation of radiation-induced gene expression alteration through pathway analysis

Cytoscape plugged ClueGO and CluePedia was performed for functional analysis of each differential group gene sets acquired from microarray analyses. The KEGG, REACTOME, and WikiPathways databases were applied for enrichment analysis.

**Fig 5:**
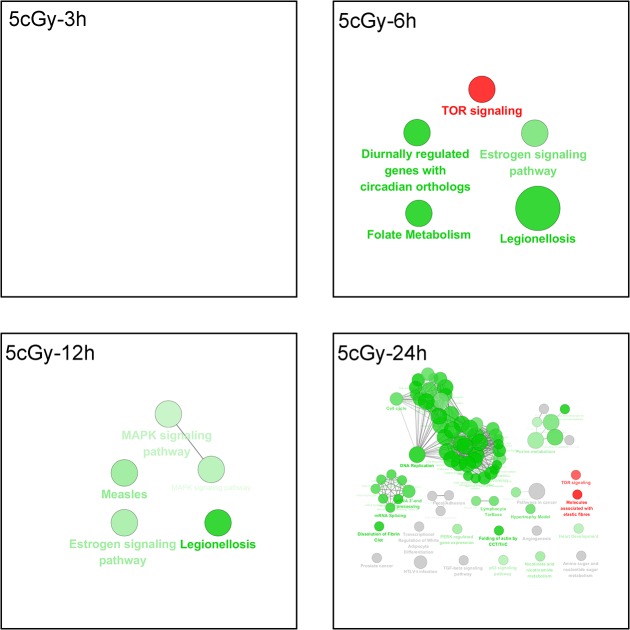
The pathway enrichment analysis results of the 5 cGy group at different time points. The KEGG, REACTOME and WikiPathways databases in ClueGO were employed for pathway enrichment analysis of each differential group gene sets acquired from microarray analyses. The Enrichment/Depletion was tested by the two-side hypergeometric test, and the Benjamini and Hochberg false-discovery rate was set to 0.05. The green and red colors code for down-regulation and up-regulation of genes, respectively, in each group. The result for 3 h post 5 cGy radiation was not shown due to absence of significant enriched pathways.

The pathway enrichment results for low-dose (5 cGy) radiation showed insignificant enriched pathways at 3 h post 5 cGy exposure, while the TOR signaling pathway was shown to be up-regulated at 6 to 12 h post irradiation till 24 h later, and the MAPK signaling pathway was down-regulated. Up to 24 h, the majority of the enriched pathways focused on the reduction in DNA replication, cell cycle arrest, RNA metabolism and processing (data shown in Fig 5).

**Fig 6:**
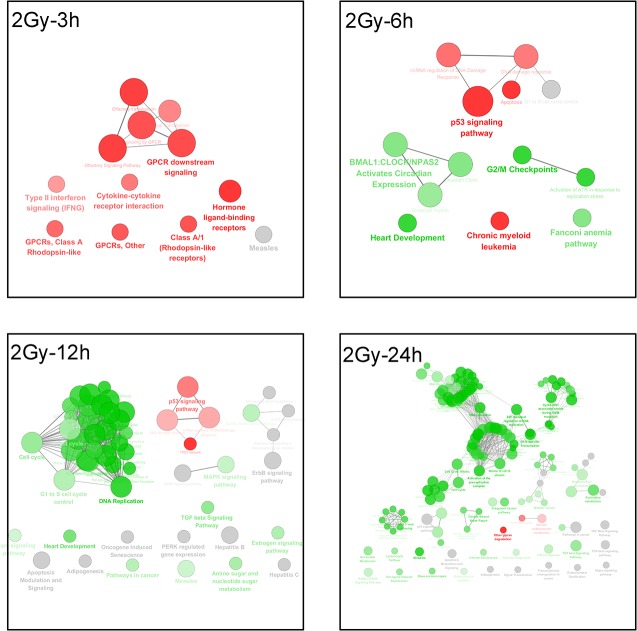
The pathway enrichment analysis results of the 2 Gy group at different time points. The KEGG, REACTOME and WikiPathways databases in ClueGO were employed for pathway enrichment analysis of each differential group gene sets acquired from microarray analyses. The Enrichment/Depletion was tested by the two-side hypergeometric test, and the Benjamini and Hochberg false-discovery rate was set to 0.05. The green and red colors code for down-regulation and up-regulation of genes, respectively, in each group.

The high radiation dose (2 Gy) led to different cellular responses. At the early stage, most up-regulated genes were involved in G protein-coupled receptors (GPCR) downstream signaling, cytokine-cytokine receptor interaction and interferon gamma signaling pathway at 3 h post irradiation. As early as 6 h post irradiation, some genes were involved in promotion of the p53 signaling pathway and responded to DNA damages, either through apoptosis or miRNA, and also slowed down cell replication via controlling cell cycle checkpoints. At later time points from 12 to 24 h post irradiation, more and more resources were spent on the negative regulation of DNA replication and cell cycle, particularly at 24 h post irradiation, and the DNA double-strand break repair term indicated repair in order to rescue the cells from the X-ray induced damages (data shown in Fig 6).

**Fig 7:**
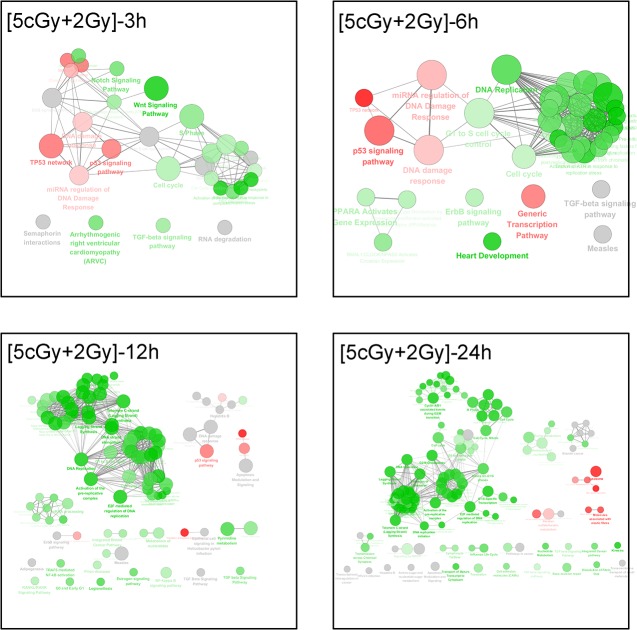
The pathway enrichment analysis results of the (5 cGy +2 Gy) group at different time points. The KEGG, REACTOME and WikiPathways databases in ClueGO were employed for pathway enrichment analysis of each differential group gene sets acquired from microarray analyses. The Enrichment/Depletion was tested by the two-side hypergeometric test, and the Benjamini and Hochberg false-discovery rate was set to 0.05. The green and red colors code for down-regulation and up-regulation of genes, respectively, in each group.

Interestingly, the (5 cGy + 2 Gy) irradiation invoked different pathways when compared to the 2 Gy irradiation at corresponding time points. The fundamental discrepancy was that most of the cellular events for the (5 cGy + 2 Gy) group were initiated after those for the 2 Gy group. For example, the p53 signaling pathway or network, miRNA regulation of DNA damage response, cell cycle terms were initiated as early as 3 h post exposure, and extended to 6 or 12 h. Moreover, some of pathways involving cell cycle control or checkpoints, DNA replication or regulation would be then strengthened at later time points. Meanwhile, some special pathways appeared. For examples, the Notch and Wnt signaling pathways were enriched at 3 h, and lysosome activation was enhanced at 24 h, which suggested that the cells initiated autophagic digestion to process the injured cell to avoid larger-scale damages (data shown in Fig 7).

### Identification of specific radiation-induced microRNA

**Fig 8:**
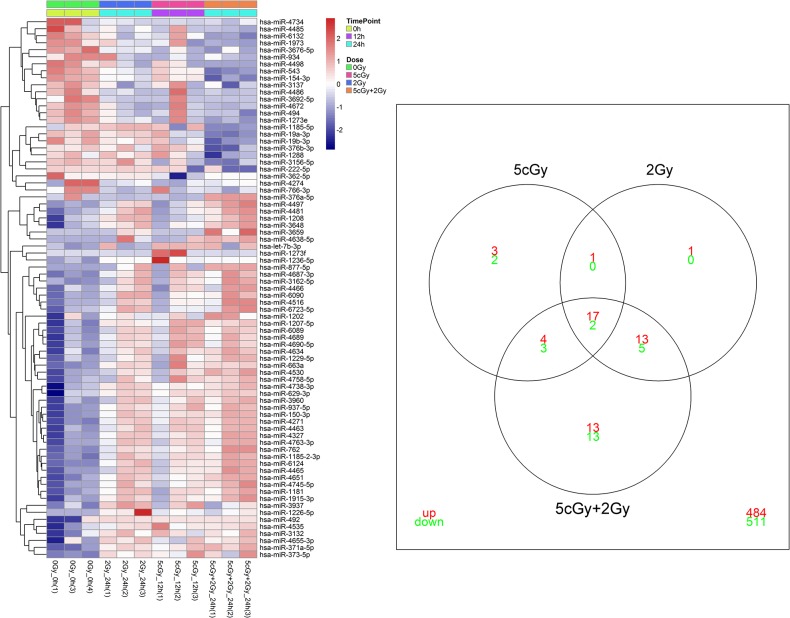
The heatmap of expressed miRNAs and Venn diagram of overlap for the three irradiation groups. The left panel shows the significantly expressed miRNAs using Bayes’ estimation in the limma package. The right panel shows the Venn plot of DEG numbers of the three groups, with green and red colors code for the numbers of down-regulated and up-regulated genes, respectively.

According to the previous microarray study, microRNA(s) were likely involved in irradiation-induced gene regulation. To investigate the potential microRNA(s), we made use of the irradiated samples from the 5 cGy group at 12 h post radiation, the 2 Gy group at 24 h post radiation, and the (5 cGy + 2 Gy) group at 24 h post radiation. Using Bayes’ estimation in the limma method, we found 32, 39 and 70 significantly expressed microRNAs in the 5 cGy, 2 Gy and (5 cGy + 2 Gy) groups, respectively (Fig 8). The majorities of differentially expressed microRNAs were summarized in Table 3, and were also identified by miRNA qPCR. The RT-PCR data indicated that over 83% (2 out of 24 genes and time points, e.g., hsa-miR-492 in 5 cGy and 2 Gy groups) of the ratios generated by microarray hybridizations were valid. Three microRNA databases (miRBase, TargetScan and miRGen) were used to identify the mRNAs, and to analyze the gene functions and pathways of the predicted mRNA.

**Table 3:** Significantly expressed microRNAs for different irradiation groups and qPCR validation.

microRNA	Group	Microarray relative change (log2 Fold change)	qPCR relative change
hsa-let-7b-3p	5 cGy	1.655	1.041 ± 0.200
hsa-let-7b-3p	2 Gy	**-**	1.082 ± 0.020
hsa-let-7b-3p	5 cGy + 2 Gy	**-**	0.933 ± 0.448
hsa-miR-1185-5p	5 cGy	-0.146	0.883 ± 0.084
hsa-miR-1185-5p	2 Gy	-1.381	0.821 ± 0.158
hsa-miR-1185-5p	5 cGy + 2 Gy	-2.821	0.718 ± 0.034
hsa-miR-1236-5p	5 cGy	3.841	1.014 ± 0.300
hsa-miR-1236-5p	2 Gy	**-**	1.606 ± 1.194
hsa-miR-1236-5p	5 cGy + 2 Gy	**-**	1.280 ± 0.142
hsa-miR-222-5p	5 cGy	**-**	0.653 ± 0.044
hsa-miR-222-5p	2 Gy	**-**	0.528 ± 0.070
hsa-miR-222-5p	5 cGy + 2 Gy	-5.431	0.377 ± 0.055
hsa-miR-3659	5 cGy	0.039	1.423 ± 0.523
hsa-miR-3659	2 Gy	1.615	1.675 ± 0.249
hsa-miR-3659	5 cGy + 2 Gy	4.421	2.184 ± 0.360
hsa-miR-4535	5 cGy	2.097	1.624 ± 0.522
hsa-miR-4535	2 Gy	1.717	2.254 ± 0.864
hsa-miR-4535	5 cGy + 2 Gy	1.928	2.734 ± 0.895
hsa-miR-492	5 cGy	4.076	0.853 ± 0.042
hsa-miR-492	2 Gy	4.167	0.870 ± 0.184
hsa-miR-492	5 cGy + 2 Gy	4.132	1.158 ± 0.336
hsa-miR-877-5p	5 cGy	1.782	1.235 ± 0.394
hsa-miR-877-5p	2 Gy	2.715	1.341 ± 0.434
hsa-miR-877-5p	5 cGy + 2 Gy	5.541	2.282 ± 0.617

**Fig 9:**
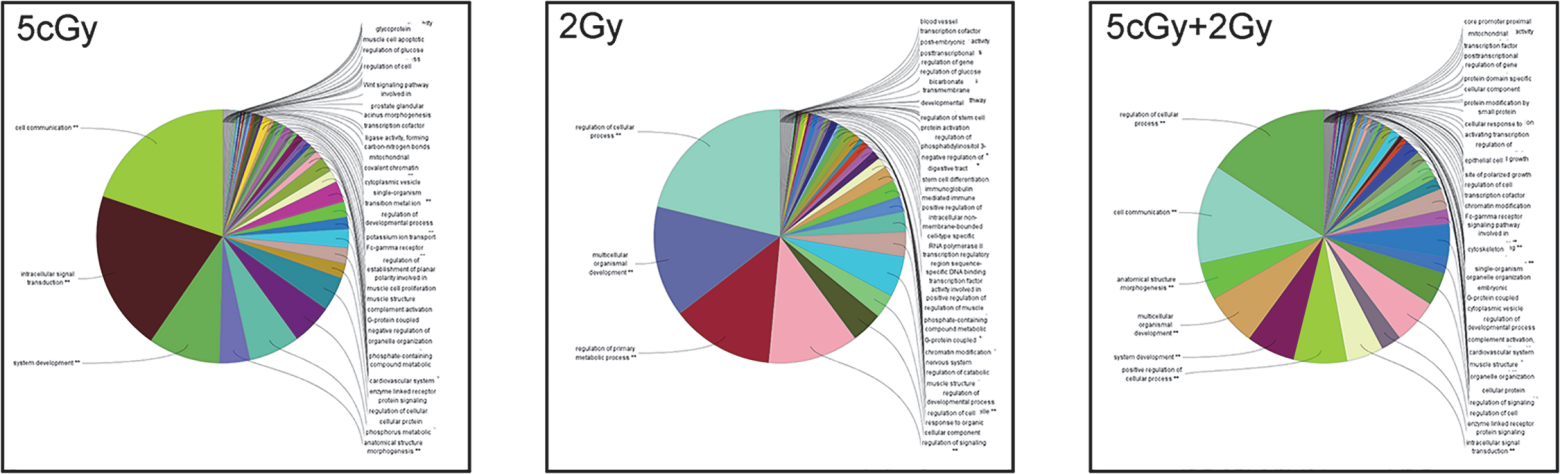
The pie charts showing the predicted mRNA enrichment GO terms for the three irradiation groups. The mRNAs predicted using three microRNA databases were input into ClueGO plugged in Cytoscape for enrichment analysis for gene ontology. The Cellular Component, Biological Process, Molecular Function and Immune System Process GO terms were selected for this analysis. The two-sided hypergeometric test was used in the statistical inference, and the Benjamini-Hochberg method was applied in p value correlation. The adjusted p-value threshold was set to 0.001.

When we compared the mRNA GO terms predicted for the three groups, the low-dose 5 cGy group showed that most GO terms focused on cell communication, intracellular signal transduction and system development (Fig 9). Meanwhile, the enrichment pathway also indicated that these predicted mRNAs were dominnat in translation, chromosome maintenance, meiotic recombination and Wnt signaling pathways and other metabolic pathways or terms (Fig 9). On the other hand, however, a high-dose (2 Gy) radiation induced many genes involved in regulation of cellular processes and primary metabolic processes, multicellular organismal development and regualtion of signaling (Fig 9). These were also confirmed by the pathway results (S10 Fig). The more notable results were the EGFR pathway, eukaryotic translation elongation and RNA process, metabolism of vitamins and cofactors, calcium and Wnt signaling pathways (S11 Fig). The RAR group, with 5 cGy priming dose and 2 Gy changelling dose, showed that the GO results were most likely a combination of the individual results for the 5 cGy and 2 Gy groups. It combined both of their GO terms, comprising cell communication, intracellular siganl transduction, including most of the enriched results for the 5 cGy case, and also regulation of cellular processes for the 2 Gy group. Furthermore, the enrichment results also showed complicated pathways, including EGFR signaling, insulin signaling, exon junction complex, metabolic and RNA and DNA processes (S12 Fig).

## Discussion

RAR is a non-targeted effect that does not require direct irradiation of the cell nucleus [60]. It occurs when a preceding low priming dose decreases the biological effectiveness of a subsequent high challenging dose. For clinical radiotherapy, RAR can potentially be exploited to avoid or to reduce the excessive damages on normal somatic cells inflicted by the radiation used to kill the tumor cells. Until now, the mechanisms underlying RAR are still largely obscure. Most previous researches focused on a single gene or protein that was linked to RAR, which was not entirely accurate and precluded a holistic view on the process. In this study, we aimed to examine the extensive radiation responses at the entire transcriptome level for a low dose (5 cGy), a high dose (2 Gy) and a combination of 5 cGy priming dose and 2 Gy challenging dose (5 cGy + 2 Gy) on irradiated AG01522 cells, and to identify RAR or dose specific factors.

Moreover, the results in previous studies on RAR were not always consistent, which might depend on the cell type, the dose range and time interval between exposures [45]. As such, all critical factors including the priming dose, challenging dose and the time interval between exposures were optimized, which were described in the previous text.

The present results clearly demonstrated that pre-exposure to a low-dose radiation changed the cellular responses at the early stage. From the GO and pathway results, we found that the defense mechanisms triggered by the 5 cGy irradiation at least 6 to 12 h earlier than those triggered by the 2 Gy irradiation. Therefore, the (5 cGy + 2 Gy) group benefited more from the 5 cGy dose which stimulated acute-phase responses. Our time-course data also revealed that the radiation induced cellular responses within as early as 3 to 6 h, including cell cycle arrest, proliferation, DNA and RNA metabolism, replication and transduction. These agreed with the previous finding on radiation-induced bystander effect that the early response within 2 h involved p53-induced or regulated genes in fibroblast cells irradiated with a dose of 2 Gy [42].

Based on gene ontology or pathway analysis, the GPCRs were activated within 3 h post irradiation for both the 5 cGy and 2 Gy groups. Ionizing radiation is known to induce expression of cytokine receptors and G proteins [61], which was confirmed by our present results showing up-regulation of G–protein-mediated signaling pathway. The activation of G proteins and cytokine receptors could be explained in terms of mitogenic/proliferative signaling to promote cellular survival under genotoxic stress. At downstream to those receptors and G proteins signaling, phospholipase C-g (PLC-g), protein kinase C (PKC)/Ras/Raf network would probably be involved in the proliferative response [62, 63]. Moreover, from any group of early response after irradiation, the olfactory pathway or terms had been enriched, and neural cell adhesion molecule (NACM) interaction term had also been found in GSEA. Based on the GSEA gene family database, we found some core genes (ARTN, GDNF) in the NACM interaction term including cytokines and growth factors. Furthermore, other critically enriched genes included the olfactory receptors (COL4A5, COL4A4, COL4A3 and COL9A3) and also GPCRs, which suggested that olfactory factors might trigger radiation response that promoted the downstream effects. Previous research also indicated that radiochemical formation of ozone and free radicals might stimulate olfactory receptors in radiation processes [64, 65].

Several types of cellular responses to low-dose ionizing radiation, such as adaptive response and bystander effect, are very different from their high-dose counterparts. Accumulated evidence has also shown that the biological effects are not linearly related to the dose. The predominant functional groups responding to low-dose radiation are those involved in cell-cell signaling, signal transduction, development and DNA damage responses. At high doses, the responding genes are involved in apoptosis and cell proliferation [41]. In the present study, our results showed that 5 cGy irradiation induced minor DNA lesions which did not extensively damage the cells. Instead, the injury accelerated signaling transduction in the downstream to stimulate or promote cell repair. On the other hand, a high dose of 2 Gy undermined normal cellular processes irreversibly, despite the initiation of cell cycle arrest and down-regulation of DNA replication. However, application of a small priming dose of 5 cGy benefited the cells when they responded to the damages through cell cycle control, DNA replication blocking, nucleotide metabolism, lysosome and apoptosis, with an enhancement in cell repair and survival. Our microRNA expression profiles also confirmed this observation, in that cell communication and intercellular signaling transduction could be induced for the 5 cGy, or (5 cGy + 2 Gy) irradiation groups, but not for the 2 Gy irradiation group. This result hinted at a distinction between the biological effects triggered by low-dose and high-dose irradiation. The Notch signaling pathway is important in the regulation of cell fate specification, cell proliferation and cell death. Some core enriched genes, including PSEN, NCSTN and APH-1 genes, were up-regulated in the 5 cGy group, which affected the cell fate. In the RAR group, one of the GO terms involving synthetic chemical penetration coupling and uncoupling of heat-mediated respiratory electron transport of ATP, suggested that a low-dose priming radiation might provide some extra time for getting more energy to repair the damages.

From our results (data not shown) on the microRNA confirmed and predicted database enriched in the sub-network “miRNA regulate DNA damage”, we could observe that has-let-7b-5p was also involved in the 5 cGy group, which directly or indirectly regulated abundance of genes including MYC, MDM2, CCND1, CDKN1A, GADD45A, CEBPG and SESN1.

The microarray analyses revealed that many genes responsible for regulating cell cycle and cell proliferation were responsive to alteration of let-7 levels, including cyclin A2, CDC34, Aurora A and B kinases (STK6 and STK12), E2F5, and CDK8 [66]. Subsequent experiments confirmed the direct effects of some of these genes, such as CDC25A and CDK6 [67]. Let-7 also inhibits several components of the DNA replication machinery, transcription factors, even some tumor suppressor genes and checkpoint regulators [66]. Apoptosis is regulated by let-7 as well, through Casp3, Bcl2, Map3k1 and Cdk5 modulation.

Previous studies revealed that miR-222 impaired TRAIL-dependent apoptosis by inhibiting the expression of PTEN and TMIP3 [68, 69]. This microRNA plays a crucial role in the down-regulation of PTEN and TIMP3 in several types of cancers. The tumor suppressor PTEN regulates the PI3K/AKT pathway, which is a major cell survival pathway, and plays a key role in the development of multiple drug resistance [70]. TIMP3 has been reported to activate both caspase-8 and caspase-9 [71]. In this study, we found that miR-222 was down-regulated in the (5cGy + 2 Gy) group, which might indicate the cellular status of migration and apoptosis.

In this study, we performed mRNA and miRNA microarray profiles to explore further factors which regulated the RAR in normal human fibroblasts. Taken together, our microarray data identified multiple genes that were significantly up-/down-regulated during the radiation treatments. We observed that a low-dose X-ray exposure produced an alert, which triggered and altered cellular responses to defend against subsequent high-dose induced damages, and which accelerated the cellular repair process. Furthermore, microRNA analyses also revealed that cellular communication and intercellular signaling transduction played important roles upon low-dose irradiation. We conclude that RAR benefits from the alarm mechanisms triggered by a low-dose priming radation dose.

## Supporting Information

S1 Fig Transcriptional profiles of irradiated AG 1522 cells. Patterns of changes in the transcript abundance are shown on a heatmap for a robust set of 3665 transcripts using a p value of 0.05 and fold change >1.2. For each sample, the data were normalized to the average of baseline time points. The red color represents relative increase in abundance; the blue color represents relative decrease, while the white color represents no changes. The colorful bars above the map indicate time points and radiation doses.
(TIF)

S2 Fig Enrichment of GSEA for the 5 cGy group. The two phenotypes (positive and negative correlations) for the 5 cGy group obtained from GSEA were input into Enrichment app plugged in Cytoscape to perform enrichment analyses. The red color represents phenotype 1 (positive NES score in GSEA) and the blue color represents phenotype 2 (negative NES score in GSEA). The color of the inside refers to dataset 1 and the color of the outside refers to dataset 2. The size of the node (inner circle) corresponds to the number of genes in the negative-correlation phenotype (dataset 1) within the gene set, while the color of the node (inner circle) corresponds to the significance of the gene set for the negative-correlation phenotype (dataset 1). The edge size corresponds to the number of genes that overlap between the two connected gene sets. A green edge corresponds to both datasets when it is the only color edge. When there are two different edge colors, the green edge corresponds to the negative-correlation phenotype (dataset 1) while the blue edge corresponds to the positive-correlation phenotype (dataset 2).
(TIF)

S3 Fig Enrichment of GSEA for the 2 Gy group. The two phenotypes (positive and negative correlations) for the 2 Gy group obtained from GSEA were input into Enrichment app plugged in Cytoscape to perform enrichment analyses. Keys to the colors and shapes are the same as the description in the caption to S2 Fig.
(TIF)

S4 Fig Enrichment of GSEA for the (5 cGy + 2 Gy) group. The two phenotypes (positive and negative correlations) for the (5 cGy + 2 Gy) group obtained from GSEA were input into Enrichment app plugged in Cytoscape to perform enrichment analyses. Keys to the colors and shapes are the same as the description in the caption to S2 Fig.
(TIF)

S5 Fig Enrichment of GSEA for the RAR group. The two phenotypes (positive and negative correlations) for the RAR group obtained from GSEA were input into Enrichment app plugged in Cytoscape to perform enrichment analyses. Keys to the colors and shapes are the same as the description in the caption to S2 Fig.
(TIF)

S6 Fig Comparison of GO results at 3 h post irradiation for the different irradiation groups. The Gene Ontology analysis was performed by BiNGO plugged-in Cytoscape. The Biological Processes (BP), Molecular Function (MF) and Cellular Components (CC) terms were involved. To include only significant results, the FDR threshold was set to 0.05. The top 50 terms were selected, and represented as bar charts with the gene number involved in these GO terms. The adjusted p values are represented by the—log10 (adj-P value) values shown as yellow dots.
(TIF)

S7 Fig Comparison of GO results at 6 h post irradiation for the different irradiation groups. The Gene Ontology analysis was performed by BiNGO plugged-in Cytoscape. The Biological Processes (BP), Molecular Function (MF) and Cellular Components (CC) terms were involved. To include only significant results, the FDR threshold was set to 0.05. The top 50 terms were selected, and represented as bar charts with gene number involved in these GO terms. The adjusted p values are represented by the—log10 (adj-P value) values shown as yellow dots.
(TIF)

S8 Fig Comparison of GO results at 12 h post irradiation for the different irradiation groups. The Gene Ontology analysis was performed by BiNGO plugged-in Cytoscape. The Biological Processes (BP), Molecular Function (MF) and Cellular Components (CC) terms were involved. To include only significant results, the FDR threshold was set to 0.05. The top 50 terms were selected, and represented as bar charts with gene number involved in these GO terms. The adjusted p values are represented by the—log10 (adj-P value) values shown as yellow dots.
(TIF)

S9 Fig Comparison of GO results at 24 h post irradiation for the different irradiation groups. The Gene Ontology analysis was performed by BiNGO plugged-in Cytoscape. The Biological Processes (BP), Molecular Function (MF) and Cellular Components (CC) terms were involved. To include only significant results, the FDR threshold was set to 0.05. The top 50 terms were selected, and represented as bar charts with gene number involved in these GO terms. The adjusted p values are represented by the—log10 (adj-P value) values shown as yellow dots.
(TIF)

S10 Fig Western blotting analysis and validation of several key proteins from microarray results. 12, 24 and 48 h after various X-ray radiations exposure, cell lysates were collected, gel electrophoresed, and the p21WAF1, phospho-p38 MAPK, phospho-p65 NF-κB and beta-tublin were measured.
(TIF)

S11 Fig Predicted mRNA from the pathway enrichment results for the 5 cGy group. The mRNAs predicted using three microRNA databases were input into ClueGO plugged in Cytoscape for enrichment analysis of pathways. The KEGG, Reactome, Wikipathway databases were selected for this analysis. The two-sided hypergeometric test was used in the statistical inference, and the Benjamini-Hochberg method was applied in p value correlation. The adjusted p-value threshold was set to 0.05.
(TIF)

S12 Fig Predicted mRNA from the pathway enrichment results for the 2 Gy group. The mRNAs predicted using three microRNA databases were input into ClueGO plugged in Cytoscape for enrichment analysis of pathways. The KEGG, Reactome, Wikipathway database were selected for this analysis. The two-sided hypergeometric test was used in the statistical inference, and the Benjamini-Hochberg method was applied in p value correlation. The adjusted p-value threshold was set to 0.05.
(TIF)

S13 Fig Predicted mRNA from the pathway enrichment results for the (5 cGy + 2 Gy) group. The mRNAs predicted using three microRNA databases were input into ClueGO plugged in Cytoscape for enrichment analyses of pathways. The KEGG, Reactome, Wikipathway database were selected for this analysis. The two-sided hypergeometric test was used in the statistical inference, and the Benjamini-Hochberg method was applied in p value correlation. The adjusted p-value threshold was set to 0.05.
(TIF)

S1 Table mRNA qPCR Validation Primer Sets. (XLSX)

S2 Table microRNA qPCR Validation Primer. (XLSX)

S3 Table Verification of Gene Expression Changes in AG1522 12 h and 24 h after Irradiation. (XLSX)
