# Supplementary figures and images for: Gene Profiling Characteristics of Radioadaptive Response in AG01522 Normal Human Fibroblasts
Source: PLoS One. 2015 Apr 17;10(4):e0123316. doi: 10.1371/journal.pone.0123316 (PMC4401551; doi:10.1371/journal.pone.0123316)

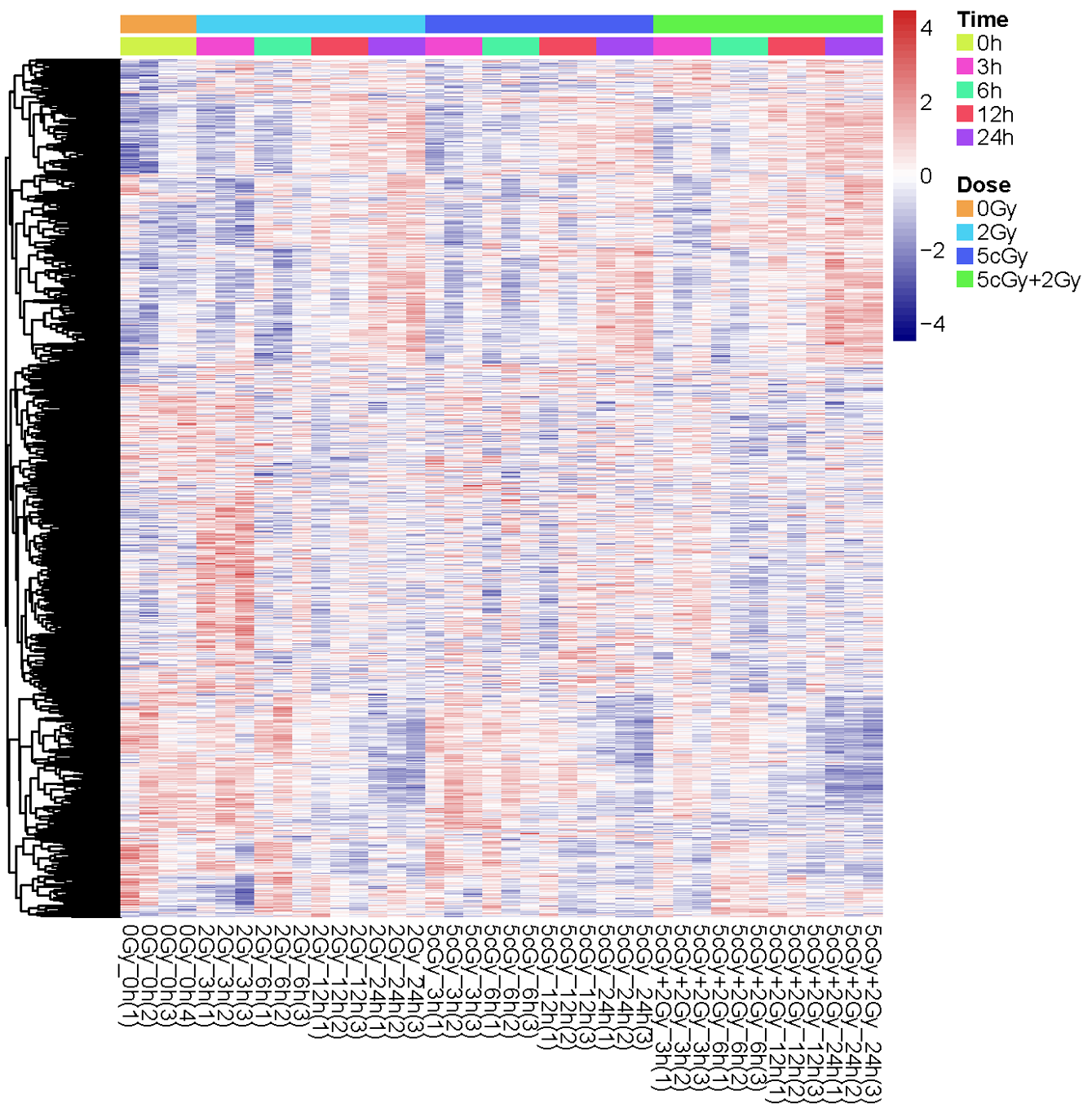

Supplement: S1 Fig — Patterns of changes in the transcript abundance are shown on a heatmap for a robust set of 3665 transcripts using a p value of 0.05 and fold change >1.2. For each sample, the data were normalized to the average of baseline time points. The red color represents relative increase in abundance; the blue color represents relative decrease, while the white color represents no changes. The colorful bars above the map indicate time points and radiation doses. (TIF) [file pone.0123316.s001.tif]

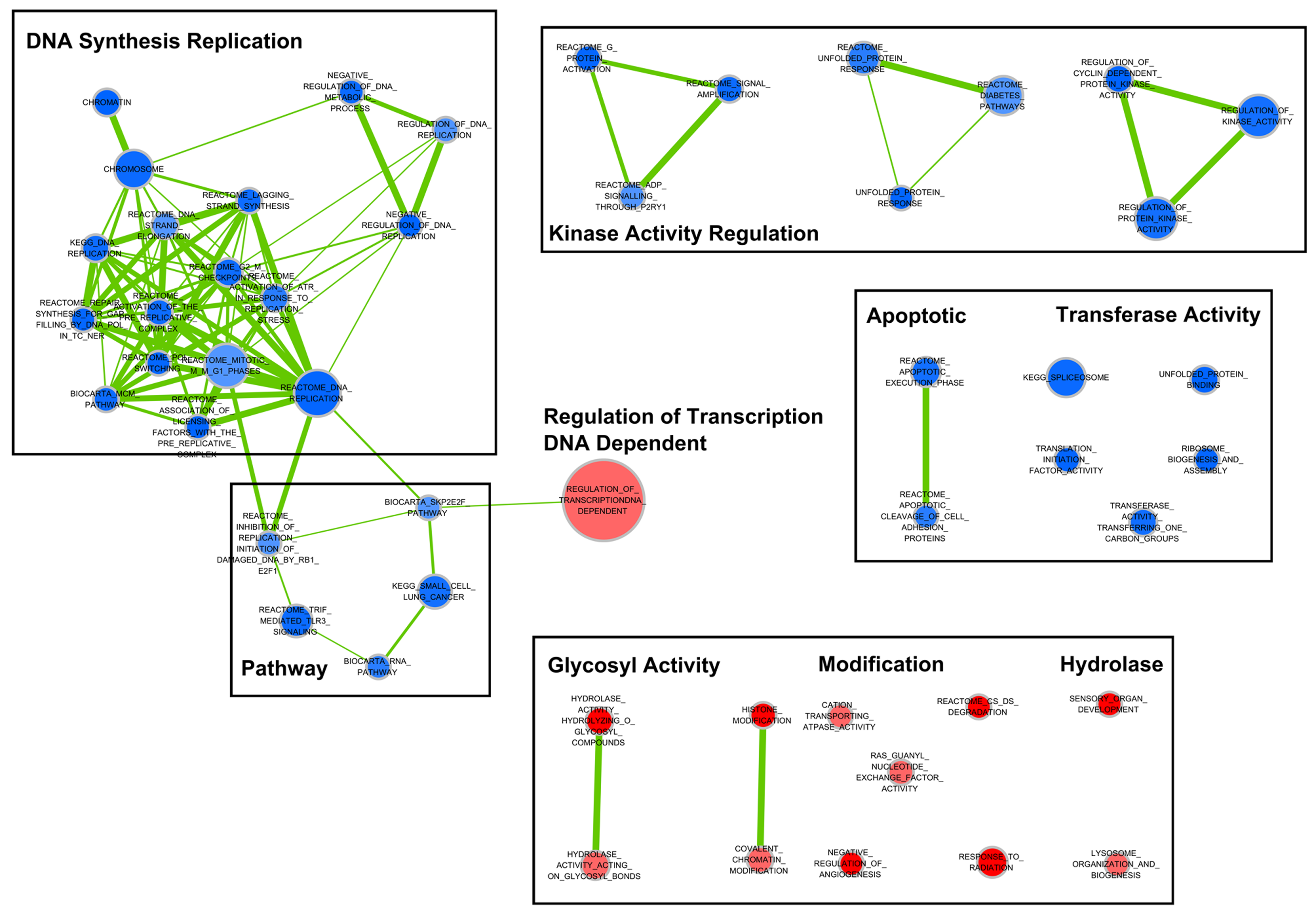

Supplement: S2 Fig — The two phenotypes (positive and negative correlations) for the 5 cGy group obtained from GSEA were input into Enrichment app plugged in Cytoscape to perform enrichment analyses. The red color represents phenotype 1 (positive NES score in GSEA) and the blue color represents phenotype 2 (negative NES score in GSEA). The color of the inside refers to dataset 1 and the color of the outside refers to dataset 2. The size of the node (inner circle) corresponds to the number of genes in the negative-correlation phenotype (dataset 1) within the gene set, while the color of the node (inner circle) corresponds to the significance of the gene set for the negative-correlation phenotype (dataset 1). The edge size corresponds to the number of genes that overlap between the two connected gene sets. A green edge corresponds to both datasets when it is the only color edge. When there are two different edge colors, the green edge corresponds to the negative-correlation phenotype (dataset 1) while the blue edge corresponds to the positive-correlation phenotype (dataset 2). (TIF) [file pone.0123316.s002.tif]

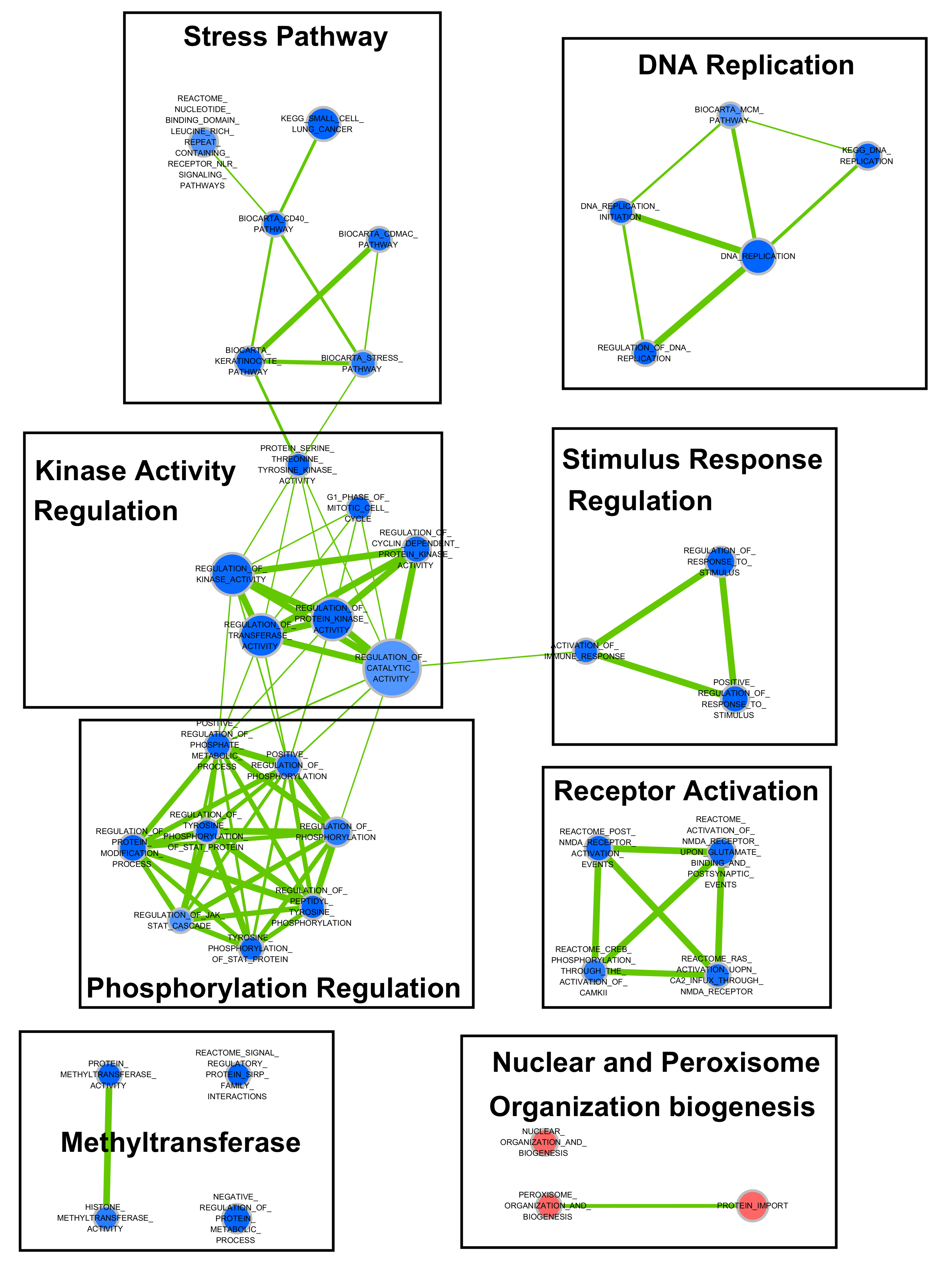

Supplement: S3 Fig — The two phenotypes (positive and negative correlations) for the 2 Gy group obtained from GSEA were input into Enrichment app plugged in Cytoscape to perform enrichment analyses. Keys to the colors and shapes are the same as the description in the caption to S2 Fig. (TIF) [file pone.0123316.s003.tif]

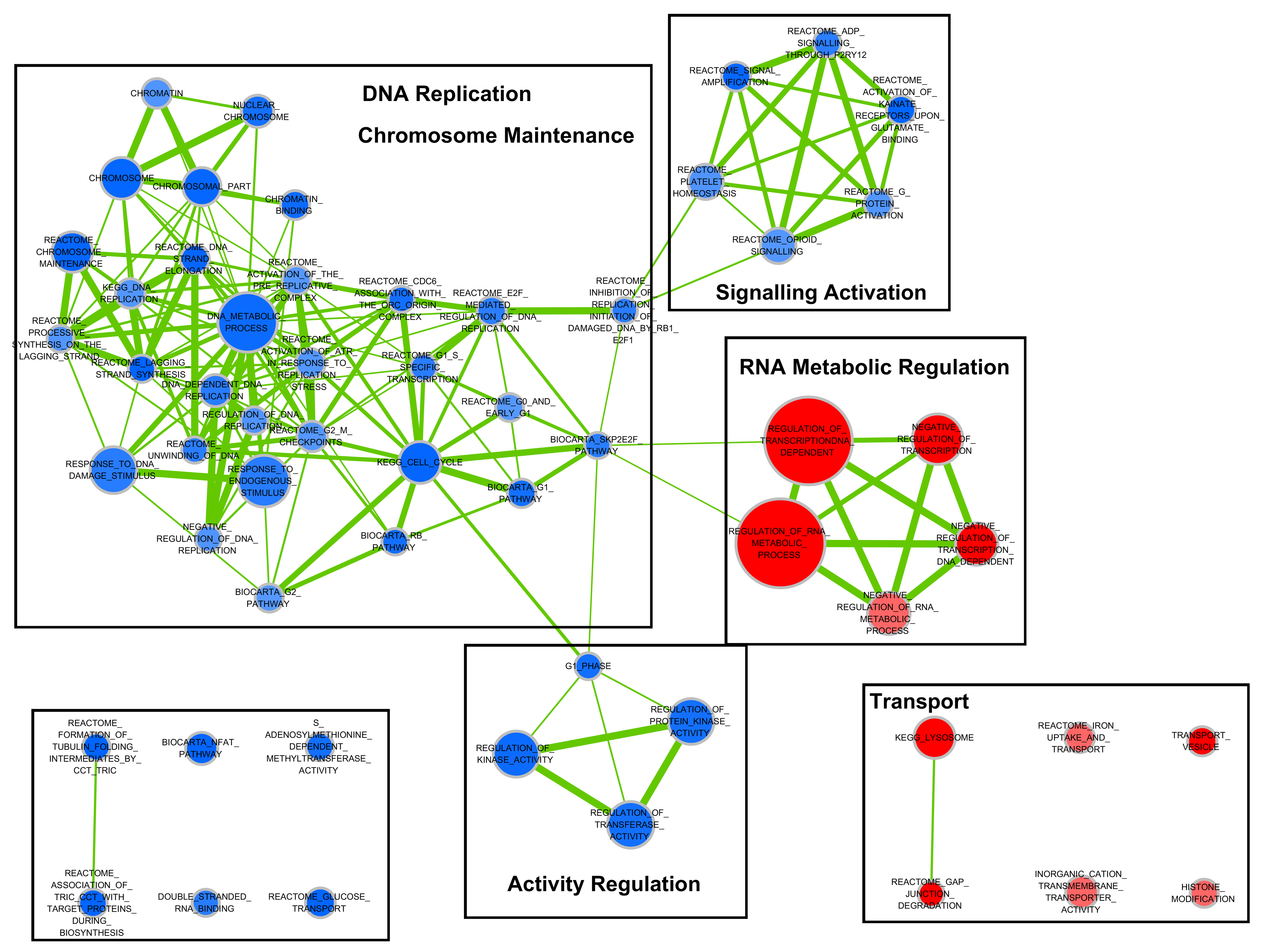

Supplement: S4 Fig — The two phenotypes (positive and negative correlations) for the (5 cGy + 2 Gy) group obtained from GSEA were input into Enrichment app plugged in Cytoscape to perform enrichment analyses. Keys to the colors and shapes are the same as the description in the caption to S2 Fig. (TIF) [file pone.0123316.s004.tif]

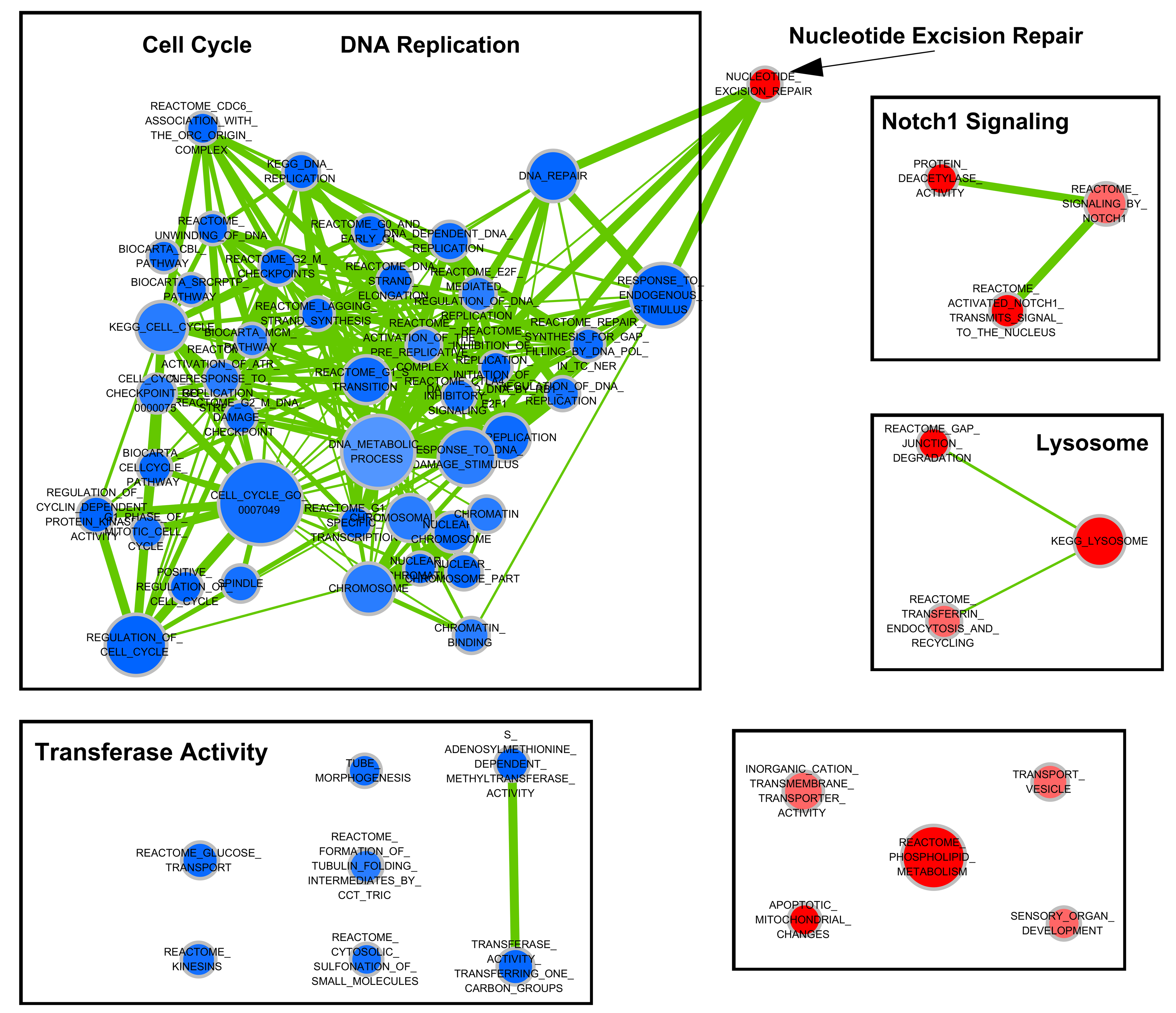

Supplement: S5 Fig — The two phenotypes (positive and negative correlations) for the RAR group obtained from GSEA were input into Enrichment app plugged in Cytoscape to perform enrichment analyses. Keys to the colors and shapes are the same as the description in the caption to S2 Fig. (TIF) [file pone.0123316.s005.tif]

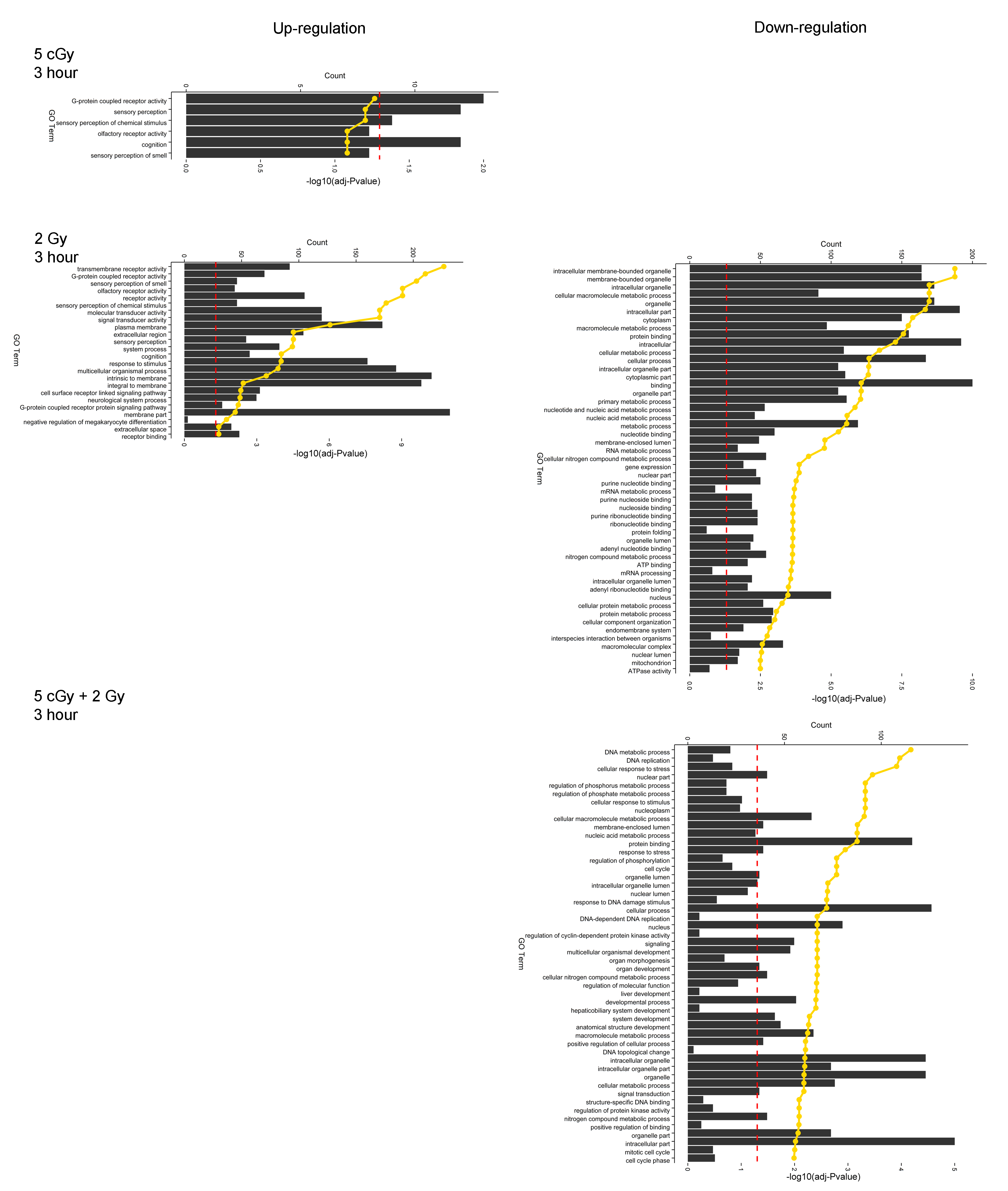

Supplement: S6 Fig — The Gene Ontology analysis was performed by BiNGO plugged-in Cytoscape. The Biological Processes (BP), Molecular Function (MF) and Cellular Components (CC) terms were involved. To include only significant results, the FDR threshold was set to 0.05. The top 50 terms were selected, and represented as bar charts with the gene number involved in these GO terms. The adjusted p values are represented by the—log10 (adj-P value) values shown as yellow dots. (TIF) [file pone.0123316.s006.tif]

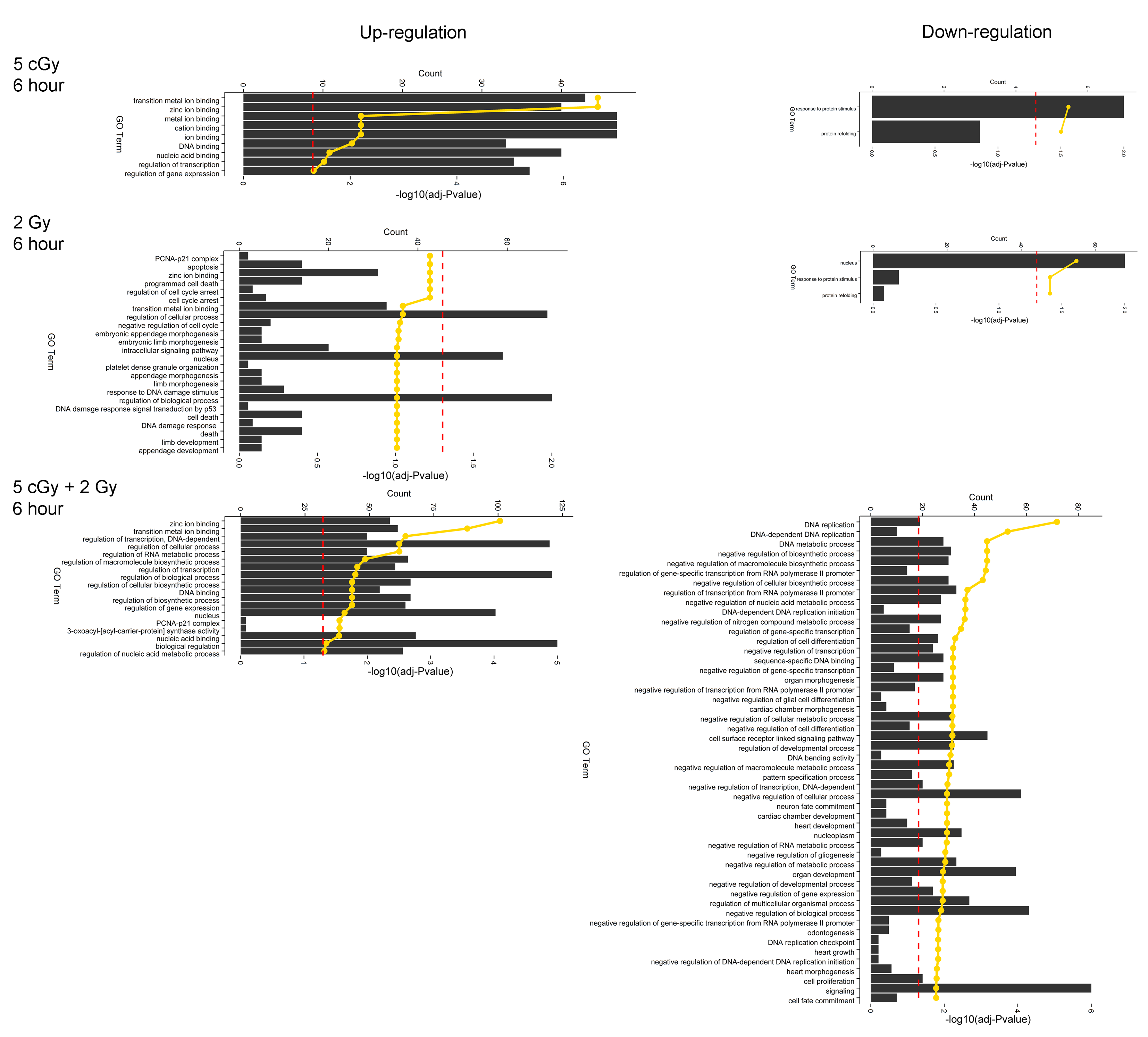

Supplement: S7 Fig — The Gene Ontology analysis was performed by BiNGO plugged-in Cytoscape. The Biological Processes (BP), Molecular Function (MF) and Cellular Components (CC) terms were involved. To include only significant results, the FDR threshold was set to 0.05. The top 50 terms were selected, and represented as bar charts with gene number involved in these GO terms. The adjusted p values are represented by the—log10 (adj-P value) values shown as yellow dots. (TIF) [file pone.0123316.s007.tif]

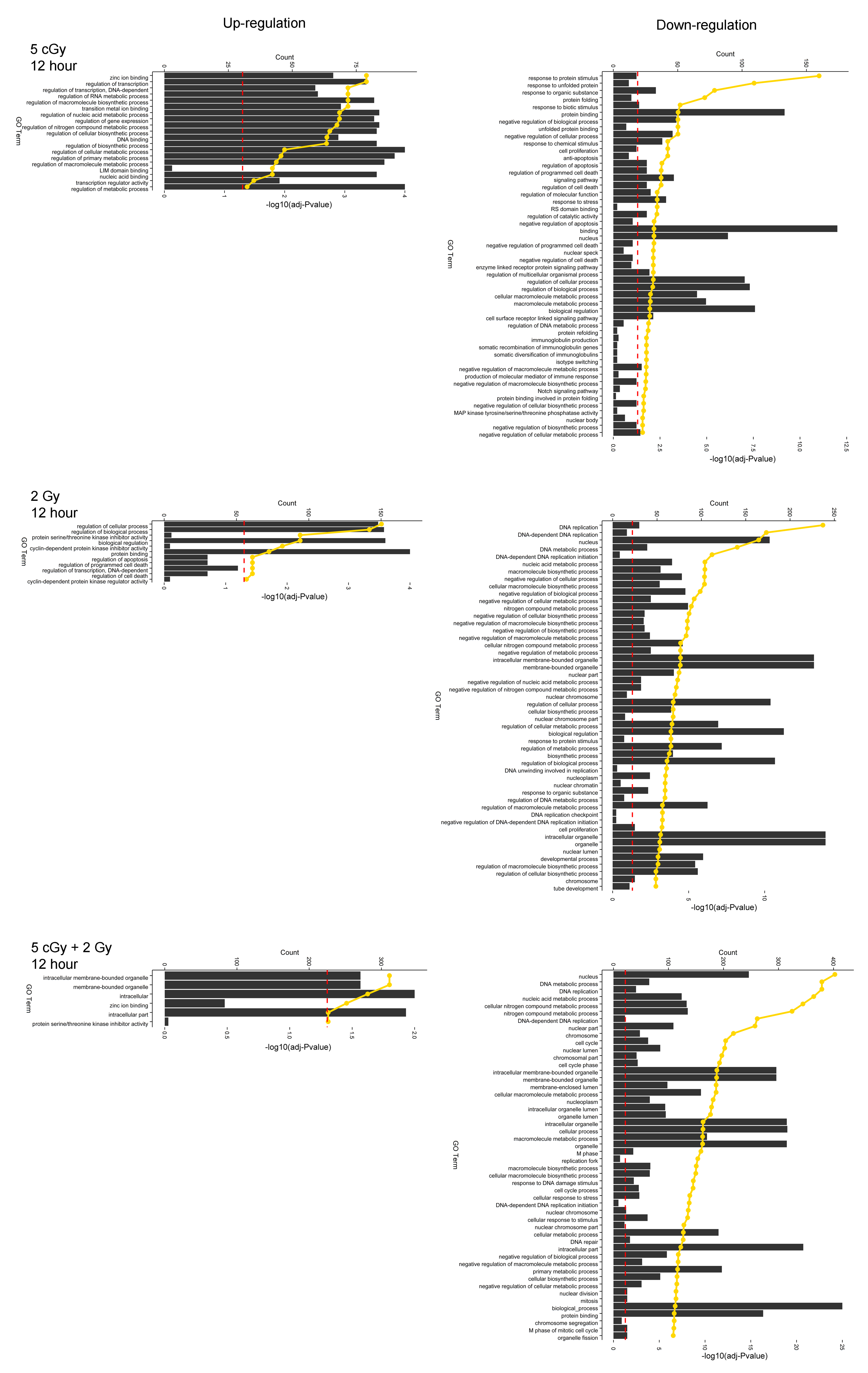

Supplement: S8 Fig — The Gene Ontology analysis was performed by BiNGO plugged-in Cytoscape. The Biological Processes (BP), Molecular Function (MF) and Cellular Components (CC) terms were involved. To include only significant results, the FDR threshold was set to 0.05. The top 50 terms were selected, and represented as bar charts with gene number involved in these GO terms. The adjusted p values are represented by the—log10 (adj-P value) values shown as yellow dots. (TIF) [file pone.0123316.s008.tif]

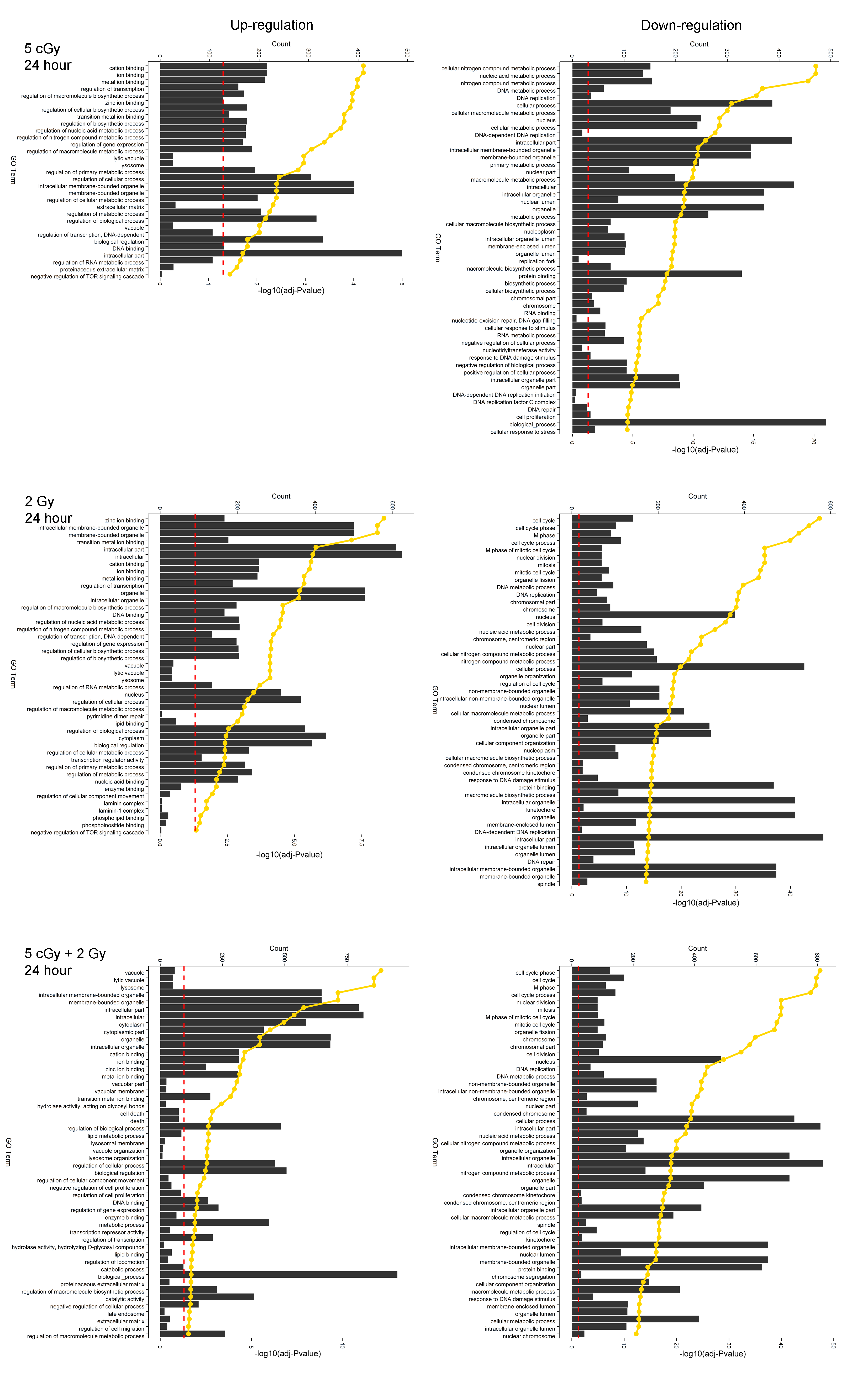

Supplement: S9 Fig — The Gene Ontology analysis was performed by BiNGO plugged-in Cytoscape. The Biological Processes (BP), Molecular Function (MF) and Cellular Components (CC) terms were involved. To include only significant results, the FDR threshold was set to 0.05. The top 50 terms were selected, and represented as bar charts with gene number involved in these GO terms. The adjusted p values are represented by the—log10 (adj-P value) values shown as yellow dots. (TIF) [file pone.0123316.s009.tif]

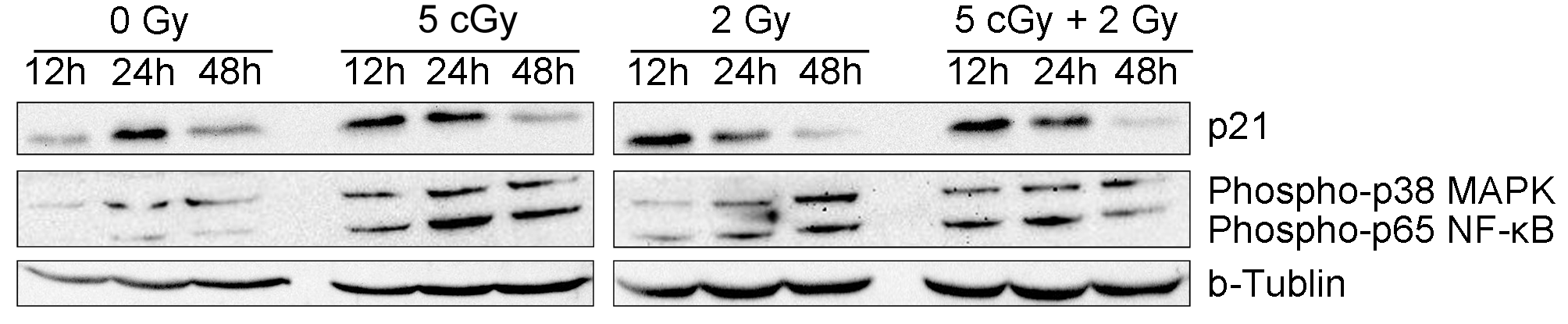

Supplement: S10 Fig — 12, 24 and 48 h after various X-ray radiations exposure, cell lysates were collected, gel electrophoresed, and the p21WAF1, phospho-p38 MAPK, phospho-p65 NF-κB and beta-tublin were measured. (TIF) [file pone.0123316.s010.tif]

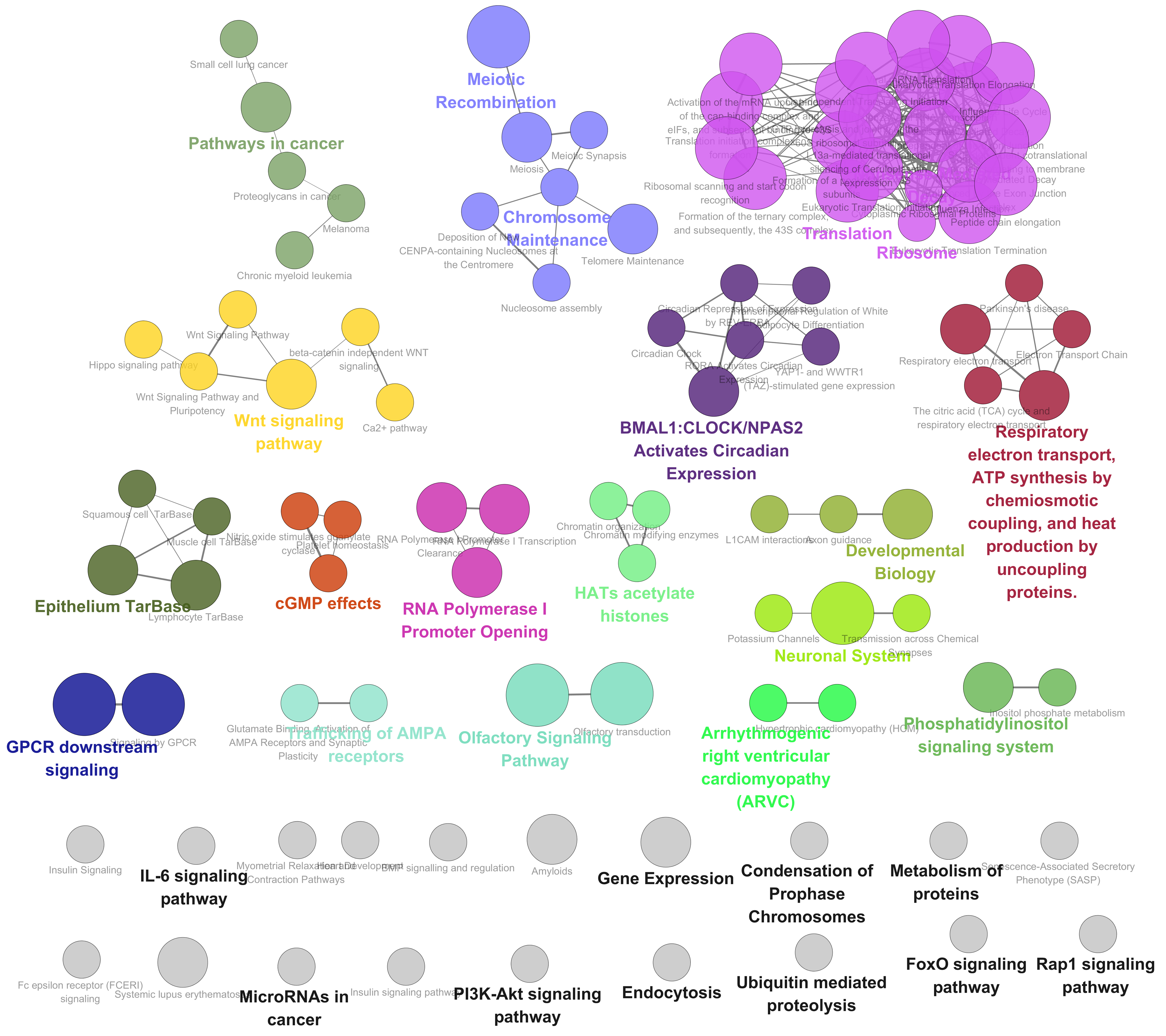

Supplement: S11 Fig — The mRNAs predicted using three microRNA databases were input into ClueGO plugged in Cytoscape for enrichment analysis of pathways. The KEGG, Reactome, Wikipathway databases were selected for this analysis. The two-sided hypergeometric test was used in the statistical inference, and the Benjamini-Hochberg method was applied in p value correlation. The adjusted p-value threshold was set to 0.05. (TIF) [file pone.0123316.s011.tif]

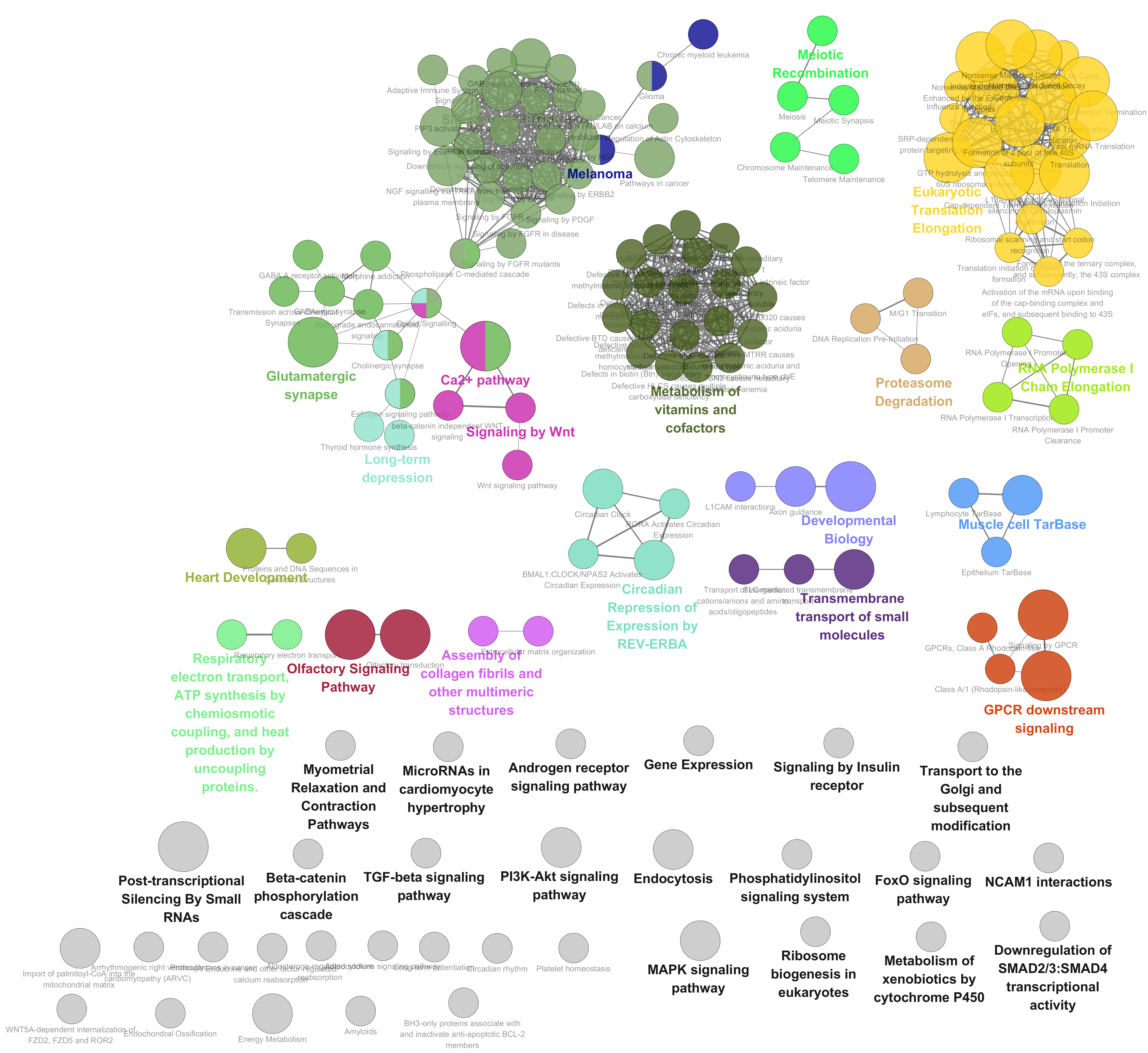

Supplement: S12 Fig — The mRNAs predicted using three microRNA databases were input into ClueGO plugged in Cytoscape for enrichment analysis of pathways. The KEGG, Reactome, Wikipathway database were selected for this analysis. The two-sided hypergeometric test was used in the statistical inference, and the Benjamini-Hochberg method was applied in p value correlation. The adjusted p-value threshold was set to 0.05. (TIF) [file pone.0123316.s012.tif]

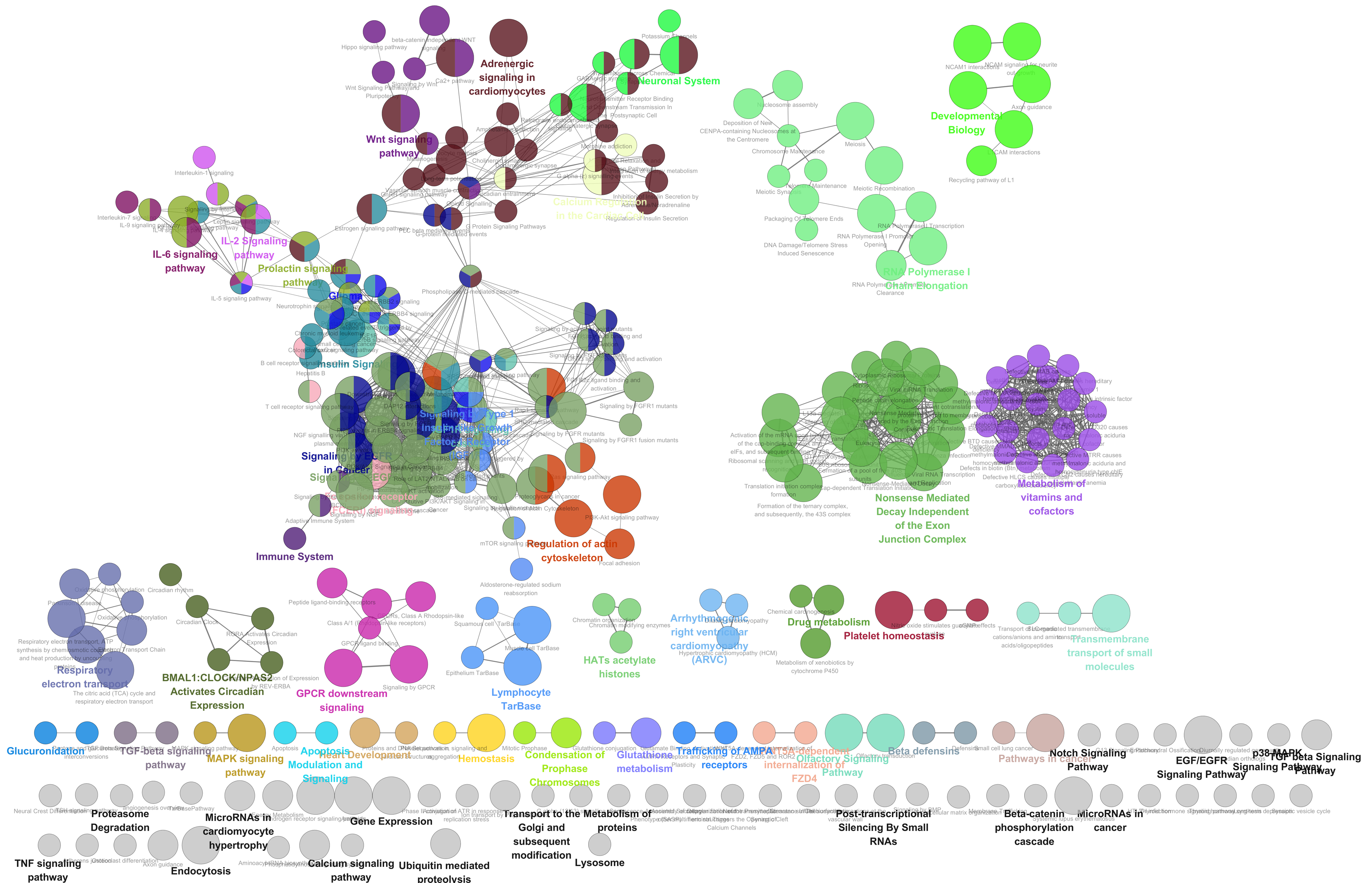

Supplement: S13 Fig — The mRNAs predicted using three microRNA databases were input into ClueGO plugged in Cytoscape for enrichment analyses of pathways. The KEGG, Reactome, Wikipathway database were selected for this analysis. The two-sided hypergeometric test was used in the statistical inference, and the Benjamini-Hochberg method was applied in p value correlation. The adjusted p-value threshold was set to 0.05. (TIF) [file pone.0123316.s013.tif]
